# Trade induced extinction risk footprints by European financial institutions

**DOI:** 10.1038/s41598-026-58778-3

**Published:** 2026-07-10

**Authors:** Siwar Ortiz-Guzman, Julien Calas, Antoine Godin, Julie Maurin, Nicolas Roques, Daniel Kieling, Tatiana Novoa Fonseca

**Affiliations:** 1 Institute for Ecological Economics, Wirtschaftsuniversität Wien, Vienna, 1020 Austria; 2 Agence Française de Développement, 5 Rue Roland Barthes, 75012 Paris, France; 3 Biotope, 25 Imp. Mousset, 75012 Paris, France; 4 Analyse Crises Transitions, Sorbone Paris Nord University, Paris, France

**Keywords:** Ecology, Ecology, Environmental sciences, Environmental social sciences

## Abstract

Biodiversity loss is increasingly recognized as a source of systemic financial risk. The financial sector enables economic activities that exert pressure on ecosystems, sometimes far from where capital is held, so institutions in one region can enable biodiversity loss elsewhere by financing seemingly harmless sectors with value-chain links to harmful activities. We establish a novel correspondence matrix linking 104 environmental pressures to 124 IUCN biodiversity threats, and combine it with the GLORIA input–output model to quantify species extinction-risk for 164 countries. Particulate matter, land-use change, and nitrogenous gases are the most damaging pressures, exerted mainly by agriculture, manufacturing, and construction. Seventy-four countries are net importers of extinction risk and twenty-three net exporters; all twelve European countries analysed are importers, with approximately 40% of their financial assets linked to extinction-risk sectors. Because these pressures are so embedded in production, a 1% reduction in the extinction-risk footprint propagates almost one-for-one through the economy: output falls by about 0.88% on average, while firms’ capacity to service debt weakens as revenues drop but interest obligations hold fixed. This exposes how structurally dependent financial systems are on nature-harming activity, supporting efforts to disclose and reduce holdings of assets linked to biodiversity loss.

## Introduction

Biodiversity loss is increasingly recognized as a source of systemic financial risk^1^. Financial regulators and academic research emphasize that the financial system is exposed to risks arising from environmental degradation, including climate change and biodiversity loss^2–4^. These risks are typically classified as physical or transition risks. This study focuses exclusively on transition risk, defined as the risk of adverse economic and financial effects resulting from policy, regulatory, or market adjustments aimed at reducing environmental harm^1,5^. Financial institutions may therefore face losses if their portfolios are concentrated in sectors exerting significant pressure on biodiversity and are affected by tightening environmental regulation^6^.

Biodiversity underpins the maintenance of ecosystem functions that sustain economic activity. For example, grasslands richer in plant species tend to exhibit more efficient biomass production, even under varying environmental conditions^7^. Soil nutrient recycling depends on plant-species diversity and particularly on the diversity of their functional traits^8^. Likewise, ecosystem functions such as biomass production, organic matter concentration, soil microbial activity, and nutrient cycling are more resistant to climate change in more biodiverse systems^9^. The degradation of biodiversity therefore affects ecological resilience and the stability of ecosystem services upon which production systems depend.

Economic activities exert environmental pressures that directly affect biodiversity and ecosystem functioning. Land artificialization, habitat destruction, fragmentation, and degradation threaten pollinator diversity and abundance^10^. Intensive agricultural practices such as monoculture, chemical inputs, and intensive ploughing reduce food resources and breeding spaces for pollinators. Pesticide use alters soil microbiological functioning by affecting nitrogen-fixing microbes, soil enzymes, and biochemical processes such as mineralization, nitrification, denitrification, and ammonification^11^. Other pressures arise from modifications to biogeochemical cycles, including excessive phosphorus discharges that cause eutrophication of water bodies and disrupt biodiversity and ecosystem functions^12^. These environmental pressures constitute the channels through which economic production translates into species extinction risk.

A growing literature has quantified biodiversity loss embodied in international trade through consumption-based footprint measures^5,13–16^. These studies show that biodiversity pressures are frequently displaced across borders, with developing countries often acting as net exporters of biodiversity loss. However, existing trade-based assessments typically rely on aggregate biodiversity footprint metrics that do not explicitly attribute species extinction risk to underlying environmental pressures^13–16^. As a result, the linkage between specific economic activities, environmental pressures, and species-level extinction risk remains only partially characterized. Yet, target 4 of the new Global Biodiversity Framework, which calls for halting extinctions of known and human-induced threatened species, could lead several countries to take measures in the coming years to reduce species extinctions.

In parallel, an emerging literature evaluates the exposure of financial institutions and central banks to biodiversity-related transition risks^3,5,6,17–19^. These studies generally measure the share of financial assets associated with high-impact sectors or biodiversity-sensitive regions. While informative, such exposure-based approaches do not quantify the macroeconomic and financial consequences of biodiversity-related policy action. In particular, they often do not estimate how reductions in biodiversity loss propagate through production systems and affect firms’ capacity to service their liabilities.

This study addresses these gaps by operationalizing species extinction risk as a function of environmental pressures and by estimating the financial-stability implications of biodiversity-loss reductions for both economic sectors and financial institutions. We use the non-normalized Species Threat Abatement and Restoration (*nSTAR*) as our biodiversity indicator^16^, which combines species’ extinction risk categories (e.g., Endangered) with species–threat impact scores derived from the IUCN Red List of Threatened Species^20,21^ for birds, mammals, and amphibians. The *nSTAR* metric provides a quantitative measure of the extent to which specific threats contribute to a species’ extinction risk.

Building on prior applications^16,21^, we link biodiversity threats to environmental pressures using the Global Resource Input-Output Assessment (GLORIA) multi-regional input–output (MRIO) model. In this framework, the *nSTAR* score captures the contribution of each environmental pressure to species extinction risk. This linkage connects economic activities to biodiversity outcomes and enables the modeling of economic impacts on extinction risk. For example, *Ateles geoffroyi* (spider monkey) faces extinction risk due to habitat loss associated with cropland expansion for coffee and cocoa production in Central America^13^. To operationalize this mapping at scale, we construct a correspondence matrix linking 104 environmental pressures to 124 IUCN-classified biodiversity threats. This procedure enables a consistent attribution of species extinction risk to economic sectors and their environmental pressures.

To assess the financial dimension of biodiversity transition risk, we combine sectoral biodiversity-loss footprints derived from the GLORIA MRIO with economic-sector liability data from the Bank for the Accounts of Companies Harmonized (BACH). BACH is a harmonised database of company financial statements, including balance sheet liabilities, interest payments, and turnover, compiled by the European Central Bank and national central banks across 12 European countries. This study provides the first multi-country assessment that moves beyond exposure metrics to quantify the economic and financial effects of biodiversity-footprint reduction. Specifically, we simulate a hypothetical 1% reduction in species extinction risk, as measured by *nSTAR*, and estimate the associated changes in economic output using the GLORIA MRIO framework. We then translate sectoral output adjustments into variations in the interest-payments-to-turnover ratio, using this indicator as a proxy for changes in firms’ debt burden and potential systemic financial vulnerability. By linking extinction-risk reduction to production adjustments and financial outcomes, the analysis provides an integrated assessment of biodiversity-related transition risk.

## Results

### Sector and pressure extinction-risk footprints

**Table 1:** Economic sector names and abbreviations based on the Nomenclature of Economic Activities (NACE) rev. 2 classification.

**NACE**	**Abbrev.**	**Full name**	**NACE**	**Abbrev.**	**Full name**
A	Agctlre	Agriculture, forestry and fishing	L	RealEst	Real estate activities
B	Mining	Mining and quarrying	M	ProfSci	Professional, scientific and technical activities
C	Mnfctng	Manufacturing	N	AdmServ	Administrative and support services
D	ElctGas	Electricity, gas, steam and air conditioning supply	O	PubAdm	Public administration and defence; compulsory social security
E	Wtr&Wst	Water supply; sewerage, waste management and remediation activities	P	Eductn	Education
F	Cnstrct	Construction	Q	Health	Human health and social work activities
G	Retail	Wholesale and retail trade; repair of motor vehicles	R	ArtEnmt	Arts, entertainment and recreation
H	Transpt	Transportation and storage	S	OtherServ	Other service activities
I	AccFood	Accommodation and food service activities	T	HouseEmp	Activities of households as employers; undifferentiated goods- and services-producing activities of households for own use
J	InfoCom	Information and communication	U	IntOrg	Activities of Extraterritorial Organizations and Bodies
K	Fin&Ins	Financial and insurance activities	X	Other	Other

**Fig. 1:**
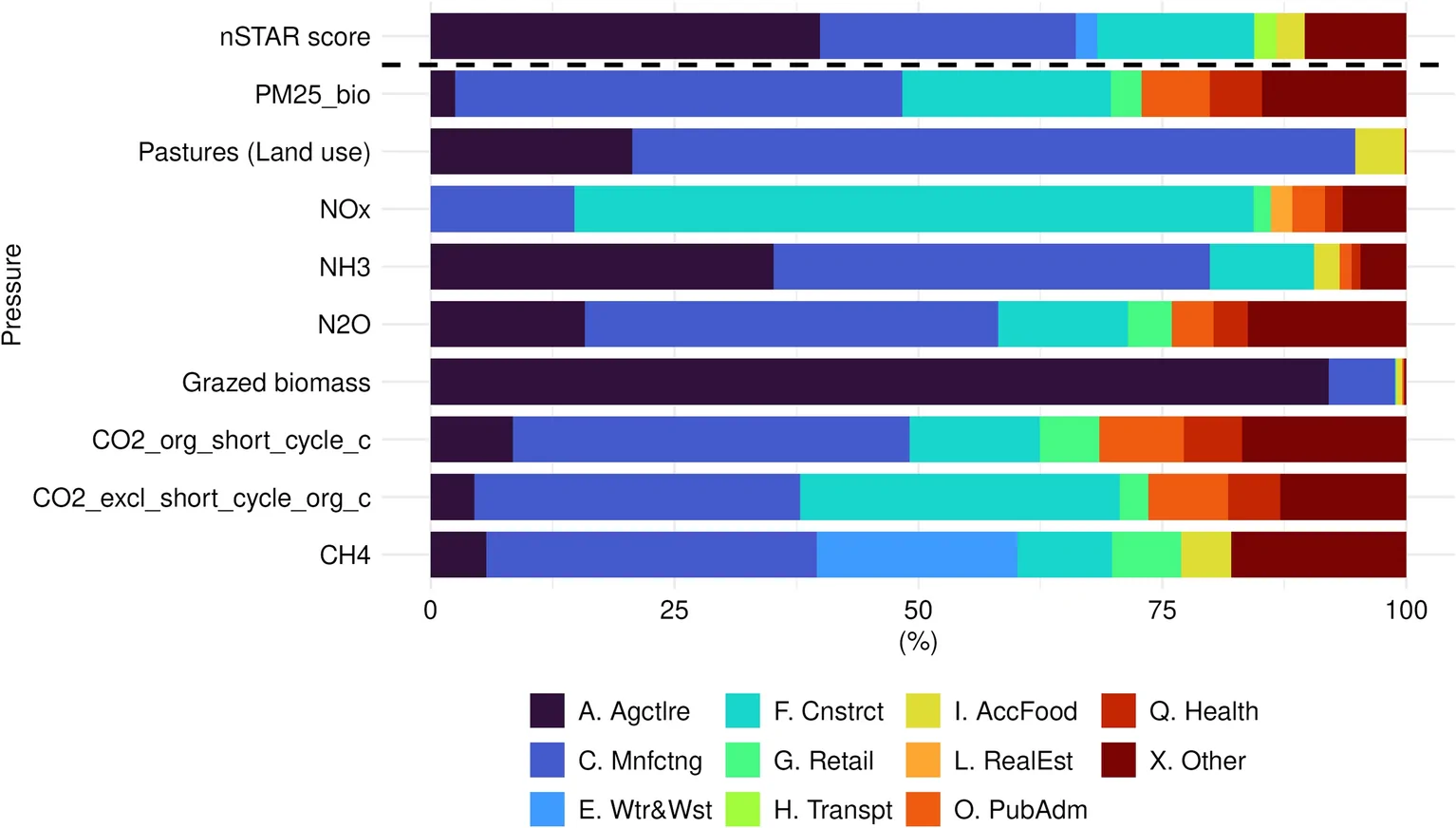
Global extinction-risk footprint by consumption sector and environmental pressure (2019). The nSTAR score, the 9 pressures that contribute most to nSTAR (aggregated across all species), and the sectors that produce them are shown. The sector “X. Other” is an aggregation of all sectors which contribution was too small to be accounted in the legend. A. Agctlre = Agriculture; C. Mnfctng = Manufacturing; E. Wtr&Wst = Water supply & waste water; F. Cnstrct = Construction; G. Retail = Wholesale & Retail trade; H. Transpt = Transportation; I. AccFood = Accomodation & Food services; L. RealEst = Real Estate; O. PubAdm = Public Administration; Q. Health = Health services.

We calculated the extinction-risk footprint of all 104 environmental pressures for all countries and economic sectors (Table 1) contained in the GLORIA MRIO. To identify which sectors and pressures have the highest footprint, we aggregated the results at the sector and pressure levels and ranked them from highest to lowest. The 9 pressures with highest *nSTAR* scores and the overall score are shown in Fig. 1 (see Table S1 for all pressures). The *nSTAR* score shown at the top represents the sum of all individual pressure scores. Furthermore, only the highest-impact economic sectors are displayed individually; the remaining sectors are aggregated into the “Other” category. Within this framework, agriculture, manufacturing, and construction account for the largest sectoral contributions to species extinction risk, contributing around 47%, 24%, and 14%, respectively. These contributions are associated with nitrogenous gases, CO$_2$, particulate matter (e.g., PM2.5), and land-use change.

Agriculture exhibits the highest *nSTAR* score. The expansion of agricultural land into natural ecosystems is widely recognized as a major driver of biodiversity loss: between 2001 and 2023, 72% of global forest loss was attributed to agricultural expansion, primarily for cattle ranching and monocultures in the tropics^22^. As shown in Fig. 1, agriculture is also the main contributor to grazed-biomass–related extinction risk. Such land-use changes are associated with habitat loss and fragmentation, which are recognized threats to biodiversity^23^.

Manufacturing contributes substantially to *nSTAR*, largely through upstream value-chain dependencies on resource extraction activities associated with pollution and habitat loss^24^. Typical activities within the manufacturing sector include production of beverages, processing of meat products, and production of cement. In some instances, meat production relies on imports originating from countries where land is converted to pasture for cattle ranching. This is reflected in Fig. 1, which shows a notable share of pasture-related extinction risk attributable to manufacturing.

The construction sector contributes to extinction risk through habitat alteration linked to infrastructure development and indirectly through pressures such as CO$_2$ emissions from building material production (e.g., cement) and fine particulate matter emissions from raw material extraction^25^. Quarrying for construction materials emits particulate matter with a diameter of 2.5 micrometers or less (PM2.5), which may affect vegetation^20^. The construction-sector footprint appears more pronounced in regions undergoing rapid urbanization. According to Seto et al^26^., infrastructure expansion driven by urban growth is expected to affect biodiversity hotspots, as many rapidly urbanizing regions overlap with areas of high species richness.

The geographic distribution of an economic sector’s contribution to species extinction risk depends on a country’s level and type of integration into international trade^16^. For example, the consumption footprint of the Agriculture sector in Europe may extend to South America and Southeast Asia due to imports of inputs such as animal feed. In contrast, the consumption footprint of some African countries may remain more concentrated within national boundaries, reflecting their role in trade networks primarily as suppliers rather than importers.

### Country extinction-risk footprints

**Fig. 2:**
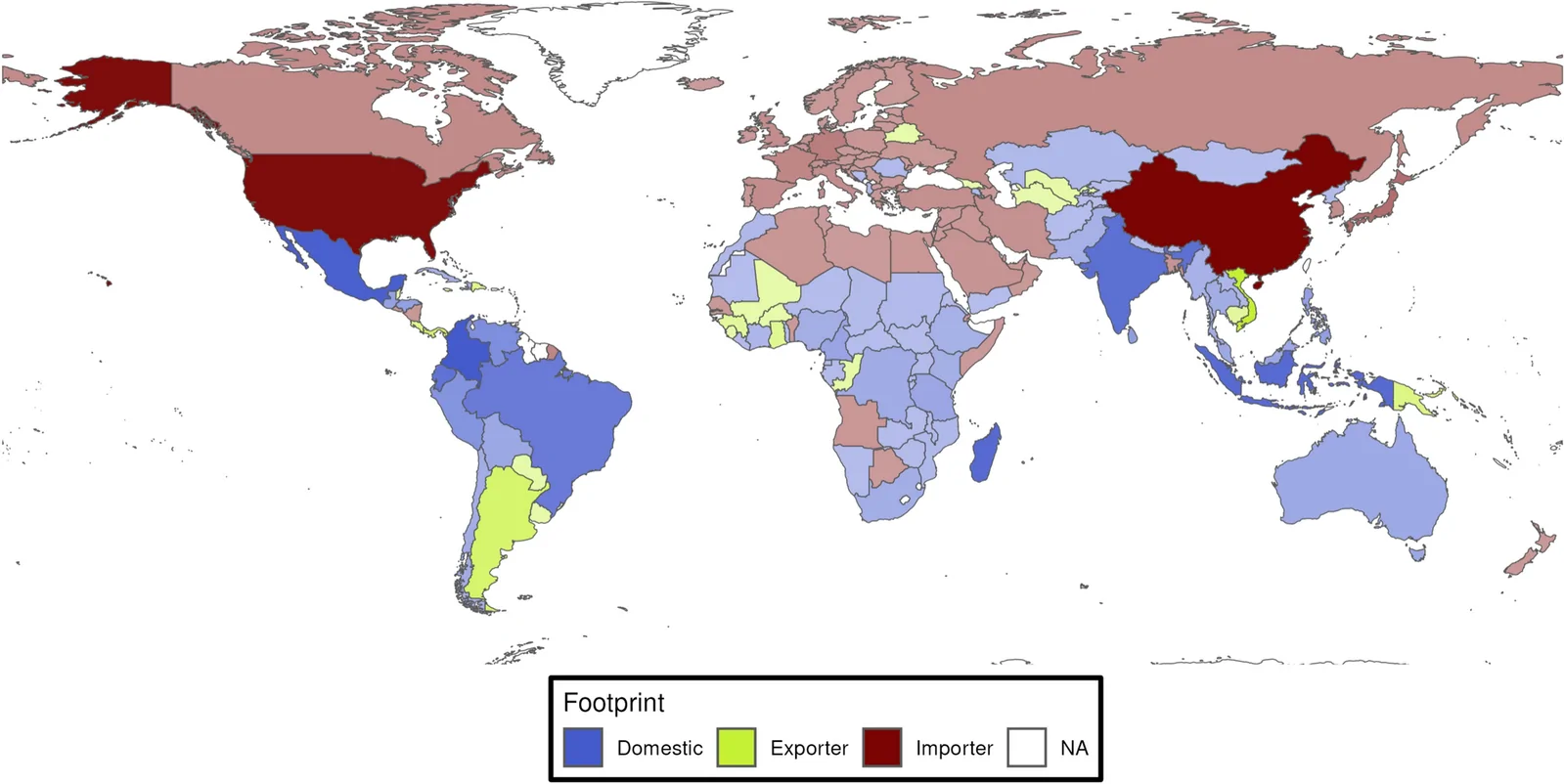
Category of extinction-risk footprint by country (2019). Each of the 164 countries in scope is colour-coded according to whether they are Net Importers (red), Net Exporters (green), or Net Domestic Consumers (blue). The darker the colour, the higher the corresponding nSTAR footprint value (imported extinction-risk footprint for countries colour-coded red, exported extinction-risk footprint for countries colour-coded green, and domestic extinction-risk footprint for countries colour-coded blue).

To characterize each country’s geographical contribution to extinction risk, we aggregated the *nSTAR* score at the country level and determined whether a country’s footprint was primarily driven by domestic consumption, imports, or exports. Figure 2 shows sixty-seven countries whose biodiversity footprint is primarily driven by domestic consumption (colour-coded blue), including Brazil, India, Indonesia, and Madagascar. Seventy-four countries are importers of species extinction risk (colour-coded red), with most located in North Africa, Europe, and North America. The United States and China exhibit the highest footprints, primarily due to imports from biodiversity-rich regions to satisfy their consumption patterns. Twenty-three countries are exporters of species extinction risk (color-coded green), among which we find agricultural and mineral commodity exporters such as Argentina and Congo.

Our results in Figure 2 differ from Irwin et al.^16^ for two main reasons. First, the reference year of the Eora MRIO employed by Irwin et al. was 2013, whereas we use GLORIA 2019, hence leading to different trade relationships (2013 vs. 2019) and different sectoral disaggregation (Eora has 26 sectors while GLORIA has 120 of them). Second, we link the environmental pressures of economic sectors to IUCN threats, whereas Irwin et al. connect economic sectors directly to IUCN threats, potentially overlooking impacts arising from the multiple pressures exerted by economic activities. The main differences are that China is categorized as importer, whereas Argentina, Uruguay, and Paraguay are categorized as exporter. Furthermore, Madagascar, Zimbabwe, Tanzania and Ethiopia are categorized as domestic consumers. For a comparison of these spatial trends using the *Potentially Disappeared Fraction* indicator see Figure S1.

The 12 analyzed European countries are importers of extinction risk footprint. This means that the consumption of food products, for example, embeds a biodiversity impact due to imports from countries whose production processes drive species extinction. The *nSTAR* score of these countries’ economic sectors is shown in Figure S2 in the supplementary material. The highest-impact sectors shown in Figure S2 are nearly identical to those shown in Figure 1, and we therefore omit it from the main text.

### European financial sector linkage with biodiversity loss

A percentage of a country’s financial assets is considered exposed to transition risk if it is linked to sectors that have a significant impact on biodiversity and whose production would therefore be affected by policies aimed at halting biodiversity loss. To measure the value of financial assets exposed to transition risk in each of the 12 analyzed countries, we calculate the share of financial liabilities held by the seven economic sectors with the highest consumption-based extinction-risk footprint (see Fig. 1, Figure S2). This was accomplished by matching BACH sector-level financial liabilities, aggregated across firms operating within each sector, to the corresponding GLORIA MRIO sectors via the NACE rev. 2 classification. To clarify the relationship between liabilities and assets, loans represent liabilities for companies, whereas they are assets for financial institutions (e.g., banks). Hence a loss in assets value would lead to solvency issues for the banks and increase the risk of bankruptcy.

**Fig. 3:**
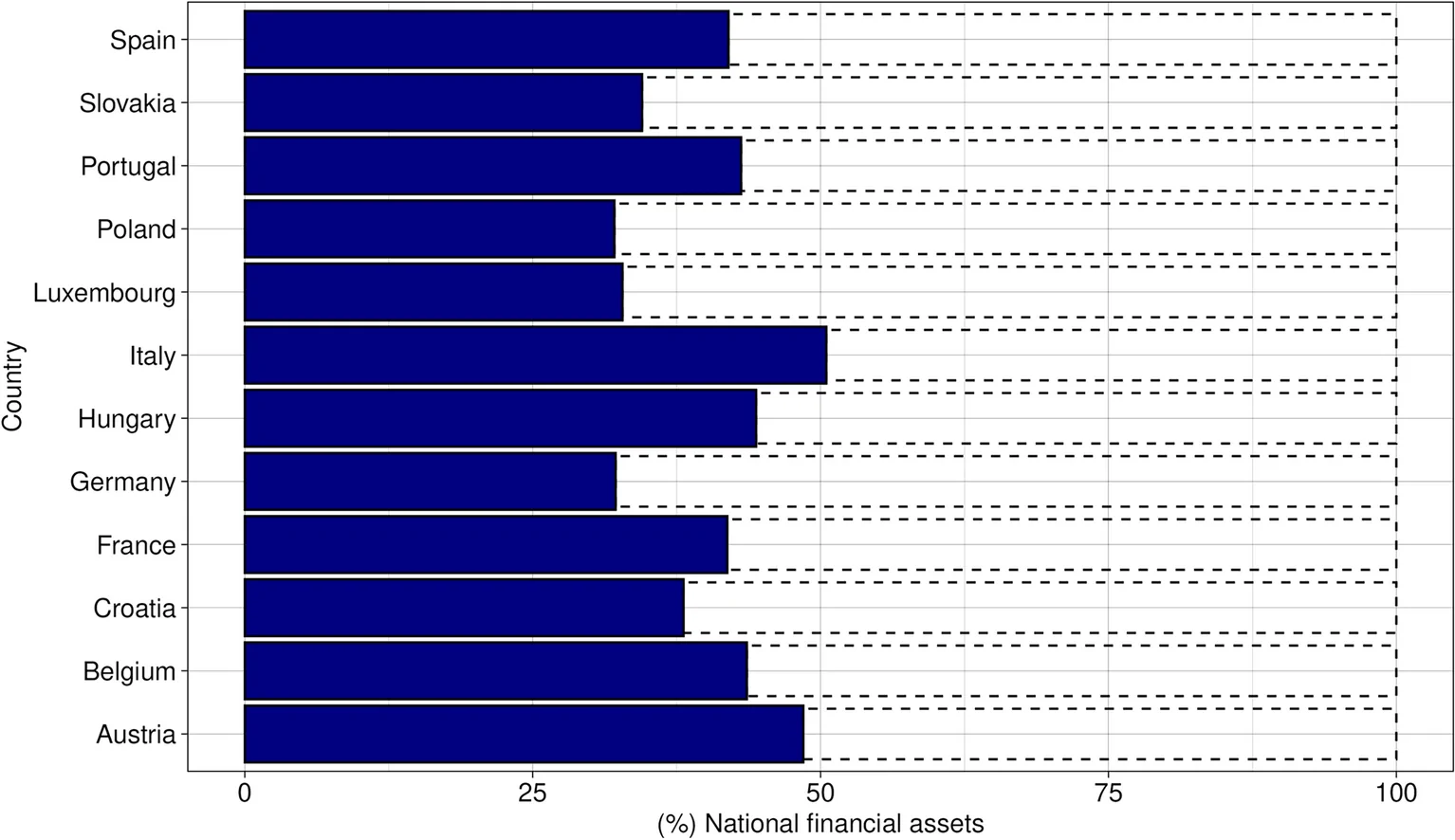
Percentage of financial liabilities of companies operating in twelve European countries linked to biodiversity loss (2019). The amounts of financial assets are aggregated by sector and the amount linked to identified critical sectors in terms of biodiversity loss (Fig. 1) are added up and compared to the total amount of financial assets.

Figure 3 shows that, on average, 40% of the financial assets in the countries analyzed would be exposed to transition risks. This implies that up to approximately 40% of total financial asset value could be affected by policies aimed at reducing Europe’s consumption–based biodiversity footprint, if the policy is based on the *nSTAR* indicator. In fact, target 4 of the 15th Conference of the Parties to the Convention on Biological Diversity proposed to “Halt Species Extinction, Protect Genetic Diversity, and Manage Human-Wildlife Conflicts” by making use of indicators such as the “IUCN Red List Index, and the nSTAR metric as a complementary indicator”^27^.

### Financial exposure to biodiversity footprint reduction

To estimate the economic effects of reducing the species extinction-risk footprint, we simulate a hypothetical policy targeting a 1% reduction in the combined consumption-based *nSTAR* footprint. The variation in final demand *Δ f* consistent with this target is derived using the fixed-coefficient structure of the MRIO framework, and the corresponding output variation is then computed via the Leontief equation.

**Fig. 4:**
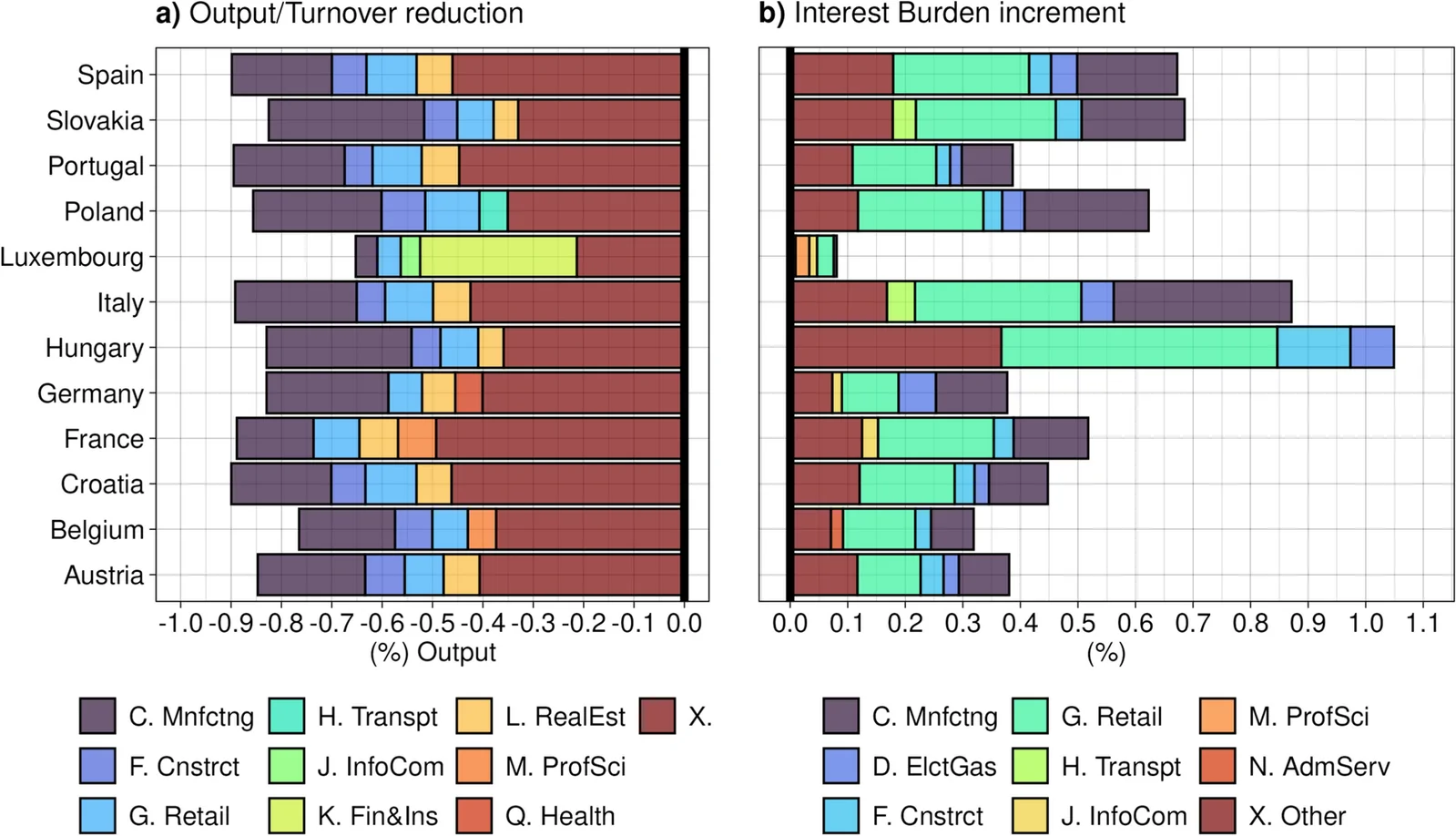
**(a)** Turnover reduction required for 1% decrease of biodiversity footprint of twelve European countries (2019). Magnitude of reduction for main affected sectors is colour-coded. The length of the bars represents the total output reduction in percentage units for each country. (**b)** Ensuing aggregate interest payment burden increase from turnover reduction. Sector-specific effects are colour coded. The total interest payment burden for each economy is represented by the length of the bars. The sector “X. Other” is an aggregation of all other sectors not displayed. **Abbreviations:** C. Mnfctng = Manufacturing; D. ElctGas = Electricity, gas, steam and air conditioning supply; F. Cnstrct = Construction; G. Retail = Wholesale & Retail trade; H. Transpt = Transportation; J. InfoCom = Information & Communication services; K. Fin&Ins = Financial & Insurance services; L. RealEst = Real Estate; M. ProfSci = Professional, scientific, and technical activities; Q. Health = Health services; N. AdmServ = Administrative & Support services.

Figure 4a shows that, on average, total national output declines by 0.88% for each country following a 1% reduction in the biodiversity footprint, reflecting the near-proportional relationship implied by the input–output structure. The most affected sectors in Europe are Manufacturing, Construction, Retail, and Real Estate (see Fig. 1), with the idiosyncratic exception of the financial sector in Luxembourg. Although agriculture shows a high *nSTAR* score in Figure 1 and its output declines by approximately 0.98%, the national-level impact remains limited because agriculture represents a relatively small share of total output in the advanced economies analyzed.

The variation in total output reduction across countries can be explained by differences in the import content of final demand. For example, Luxembourg, which exhibits a smaller output loss, is more dependent on imports than Spain or Croatia, which display larger output losses, reflecting differences in economic structure and diversification. Furthermore, the sectoral composition of output adjustments varies according to the role of sectors in European supply chains. The emergence of Financial and Insurance activities (Luxembourg), Health and Social Work activities (Germany), or Professional, Scientific and Technical activities (France and Belgium) highlights the structural specificity of these economies. Interestingly, all countries except Luxembourg show comparable reductions in Manufacturing, Construction, Wholesale and Retail Trade, and Real Estate activities, which are characteristic of advanced economies.

To assess the financial effects of the footprint reduction, we estimated the increase in the interest-payments-to-turnover ratio, where turnover is total sectoral revenue or output, for each sector resulting from reduced production. As sectoral turnover declines while nominal debt obligations and interest payments remain fixed in the short run, sectors’ capacity to service their liabilities weakens. Figure 4b shows the corresponding increase for each economic sector and country. On average, the interest burden rises by 0.6% following a 1% reduction in the biodiversity footprint. Financial effects differ from economic effects because of variations in liability structures across and within countries: Retail Trade, Manufacturing, and Construction activities appear most sensitive. These sectors are typically characterized by cash-flow mismatches, defined as timing disparities between incoming cash (e.g., sales or receivables) and outgoing cash (e.g., payments to suppliers, rent, or operating expenses), which create financing needs covered by liabilities.

## Discussion

Our results provide detailed information on the level of species extinction risk exerted by environmental pressures, economic sectors, and countries, as well as the links to twelve European countries’ financial assets. Agriculture, Manufacturing, and Construction represent the largest sectoral contributions to species extinction risk. These contributions are driven by pressures such as nitrogenous gases, CO2 emissions, particulate matter (PM2.5), and land-use change. At the country level, 74 countries are importers of species extinction risk, 23 are exporters, and 67 are primarily driven by domestic consumption. The twelve European countries analyzed are all importers of extinction-risk footprints. On average, 40% of the financial assets in these countries are linked to sectors with the highest consumption-based extinction-risk footprints. A simulated 1% reduction in sectors’ *nSTAR* scores is associated with an average 0.88% decline in total output and a 0.6% increase in the interest-payments-to-turnover ratio.

Several limitations regarding the method and data merit acknowledgment. The nSTAR metric currently covers birds, mammals, and amphibians which are the most data-complete in the IUCN Red List, making them the most defensible basis for a globally consistent metric^21^. The absence of some key groups like invertebrates and plants, likely more sensitive to certain pressures (e.g., pesticides, pollination disruption), means that our assessment understates true extinction risks and should be considered as a conservative estimate. As additional species are recorded in the IUCN Red List, the metric should progressively incorporate new taxonomic groups and become more robust^28^. Additionally, given that nSTAR scores rely on global-average data on species distributions and threats, national-level disaggregation may be subject to error arising from spatial variation in species presence and pressures. Validation using national data would be desirable but is often constrained by limited data availability; methods to reduce these biases exist^29^ and could be combined with our approach to improve national-level robustness.

Only pressures listed in the GLORIA MRIO that correspond to IUCN biodiversity threats were analysed. The matrix does not differentiate the relative contribution of each pressure to a given threat; all linked pressures are assigned equal weight, so it functions solely as a connection matrix. We consider equal weighting a transparent default under current data constraints: empirical dose–response data mapping continuous pressure levels to changes in IUCN Red List categories across species and localities do not exist at the global scale required, with the exception of GLOBIO4 with very limited pressure coverage at the community rather than species level. Given this equal weight, our results provide a lower bound on the differences between pressures (since high- and low-intensity pressures are treated equally), but they do not systematically overestimate or underestimate the ranking of one sector relative to another. One way to improve the matrix that we propose would be to supplement it in future with empirical data on the dose-response relationship, which is currently lacking at the global level.

Some threat categories, including invasive species, geological events, and human intrusions, could not be matched to GLORIA satellites, reflecting structural limitations of a database that captures emissions and land-use pressures but not other biodiversity drivers. The correspondence matrix relies on expert-assessed, literature-informed judgements rather than quantitative dose–response functions, which introduces an element of subjectivity. This approach is nonetheless consistent with precedents in the literature: Lenzen et al.^13^ employed a structurally identical binary concordance table mapping IUCN threats to economic sectors, and the widely used ENCORE database^30^ builds on analogous expert-assessed qualitative linkages between economic activities and environmental pressures. Each pressure–threat link in our matrix is documented with supporting literature, making the basis for each cell explicitly traceable.

The financial analysis is restricted to the 12 European countries covered by the BACH dataset, which limits the global applicability of the financial-stability conclusions; no comparable harmonised source of sectoral liability data with consistent disaggregation currently exists at global scale. These 12 countries nonetheless represent a significant share of European financial assets and constitute a systemically important segment of the international financial system. The methodological framework is explicitly extensible: the GLORIA MRIO covers 164 countries, and the financial liability analysis could be replicated for other regions as equivalent datasets become available – for example, through the ECB’s AnaCredit initiative or national datasets such as the Annual Financial Statistics in South Africa.

While the results are subject to the data and structural limitations described above, several considerations suggest that the main qualitative patterns are unlikely to be reversed under reasonable alternative specifications. Three sensitivity considerations deserve attention. First, the correspondence matrix captures whether a pressure–threat link exists (binary 0/1), but does not quantify the strength of that relationship; therefore, potential expert-related biases would primarily affect the allocation of extinction-risk contributions across pressure categories rather than overall sectoral rankings. Given that high-impact sectors are associated with multiple independent pressure channels, their relative prominence is unlikely to reverse under reasonable alternative specifications.

Second, although nSTAR currently covers birds, mammals, and amphibians, the dominant pressures identified–particularly land-use change and nutrient emissions–are widely recognized as cross-taxa drivers of biodiversity decline. Expanding taxonomic coverage would likely refine magnitude estimates but is unlikely to fundamentally alter sectoral ordering. Third, output and financial effects reflect proportional adjustments implied by the fixed-coefficient MRIO framework, which assumes constant marginal rates, meaning that technical coefficients, input shares, and pressure intensities are held fixed as the economy adjusts. This is a strong assumption for non-marginal policy targets: at meaningful levels of biodiversity abatement, firms would be expected to substitute inputs, restructure production processes, and face shifting relative prices, all of which would alter marginal rates. Alternative approaches such as Stock-Flow Consistent models, which allow for price responses, technological substitution, and sector-specific abatement cost curves, could modify the magnitude of estimated output and financial effects, but the identification of biodiversity-intensive sectors as economically and financially relevant would remain. The present analysis is best understood as a first-order screen that identifies exposed sectors and motivates more complex modeling, rather than as a forecast of what an actual biodiversity-aligned policy transition would entail.

This study complements the existing literature on nature-related financial risk, which has primarily relied on biodiversity extent or condition indicators^6,18,31^, by introducing an assessment based on environmental pressures’ contributions to species extinction risk^32^. By linking environmental pressures generated by economic sectors to IUCN threats, rather than connecting sectors directly to threats, the approach captures impacts arising from multiple pressures associated with economic activities. Differences with Irwin et al.^16^ reflect both the updated GLORIA 2019 database and this alternative linkage procedure. For example, China is categorized as an importer, whereas Argentina, Uruguay, and Paraguay are categorized as exporters, and Madagascar, Zimbabwe, Tanzania, and Ethiopia are categorized as domestic consumers.

Identifying the environmental pressures most responsible for species extinction risk, as well as the economic sectors that generate them, can support the design of targeted policy interventions. Rather than addressing entire sectors uniformly, the proposed method enables screening of the specific pressures within sectors that contribute most to extinction risk. Policy makers aiming to reduce species extinctions could therefore focus on modifying production processes that generate the most harmful pressures. Targeting specific environmental pressures, instead of relying solely on aggregate biodiversity indicators, also facilitates the assessment of economic and biodiversity effects of alternative strategies through models such as the one presented in this study.

For the European countries analyzed, complementary measures could include introducing biodiversity-related criteria into the Corporate Sector Purchase Programme (CSPP) of the European Central Bank. Such an approach could provide market-based incentives to reduce holdings of financial assets linked to companies with relatively high imported extinction-risk footprints. The European Union is positioned to consider such measures, as the European Central Bank has incorporated climate change and environmental degradation into its mandate^33^.

The results may also inform the development of biodiversity-related financial supervision tools. Stress-testing frameworks could incorporate sectoral extinction-risk footprints to evaluate how biodiversity-aligned transition policies may affect balance sheets under alternative scenarios. Prudential authorities could use such indicators to identify concentration risks in biodiversity-intensive sectors^33^.

For financial institutions, the analysis underscores the importance of mapping portfolio exposures to sectors associated with land-use change, pollution, and trade-linked biodiversity pressures^34^. Integrating biodiversity footprint metrics into credit risk assessment, portfolio allocation, and disclosure practices may support more informed risk management. The identification of high-footprint sectors may also assist banks in engaging with clients on transition planning and supply chain due diligence^34^. For non-financial firms, particularly those operating in agriculture, manufacturing, and construction, the findings highlight the potential financial relevance of biodiversity-related pressures embedded in global value chains. Strengthening traceability, reducing land-use-related impacts, and investing in cleaner production technologies may reduce exposure to future biodiversity-aligned policies^35^.

Aggregated metrics at the spatial level of countries or broad economic sectors provide only a first “bird’s-eye” perspective on species extinction impacts. A higher spatial resolution of environmental pressures would allow more accurate targeting of economic activities affecting biodiversity-rich ecosystems. Spatially explicit models linking consumer demand to land-use change^36,37^ could be extended to connect final demand by economic sector with financial liabilities to measure impacts on specific ecosystems. Scenario-based modeling of alternative agricultural practices^38–40^ could also be integrated into such approaches.

## Methods

We followed the steps of Irwin et al.^16^ for calculating *nSTAR* score for all countries and sectors. However, rather than linking IUCN biodiversity threat classifications directly to economic sectors, we linked them with the environmental pressures generated by those sectors. This enabled the calculation of species extinction risk at the country, economic sector, and environmental-pressure levels. We used a novel correspondence matrix, described below, to link environmental pressures to IUCN biodiversity threats. Once production-based accounts are obtained, we integrate them into the GLORIA MRIO to measure consumption-based footprints. Finally, we link economic sectors’ biodiversity footprints to their financial liabilities reported in the Bank for the Accounts of Companies Harmonized (BACH) dataset to calculate the share of financial assets connected to species extinction risk and, consequently, to transition risk. We limited our analysis to the European countries covered by the BACH dataset which are Austria, Belgium, Croatia, France, Germany, Hungary, Italy, Luxembourg, Poland, Portugal, Slovakia, and Spain.

### Correspondence matrix between MRIO environmental pressures and IUCN threats to biodiversity

The matrix is a mapping tool that directly links economic environmental pressures and biodiversity threats enabling the integration of the *nSTAR* extinction risk metric into global input–output analysis. It links 104 environmental pressures (hereafter referred to as satellites) from the GLORIA MRIO to 124 biodiversity threats defined in the IUCN Threat Classification Scheme (v3.3), as well as to the five direct drivers of change identified by IPBES (2019) (See the complete matrix). As no quantitative dose-response functions exist at the scale of 104 pressures *×* 124 IUCN threats across 164 countries and using precedent in the broader environmental footprint literature like the dependency scoring of ENCORE Database^30^ or capability matrix for ecosystem services introduced by Burkhard et al.^41^, we made the methodological choice to establish a 0/1/2 qualitative rating matrix as a starting point, which should be easy to improve through further research. It was developed by experts from the Agence Française de Développement (AFD) and Biotope, a French consulting firm specializing in biodiversity. The primary objective was to enable the attribution of extinction risk, through the calculation of the *nSTAR* score, to specific economic sectors and countries by identifying links between environmental pressures and biodiversity threats established by the scientific literature. Each pressure–threat link is supported by scientific publications that identify and characterize the relationship between them (See the matrix documentation for link justifications).

The correspondence matrix is structured as follows:

- The Y-axis lists the GLORIA satellite indicators (e.g., $CO_2$ emissions, land-use change, ammonia emissions, $PM_{2.5}$ biogenic).
- The X-axis presents the disaggregated threat categories from the IUCN (e.g., 2.1 Annual Crops, 5.3 Logging and Wood Harvesting, 9.5 Airborne Pollutants) and the IPBES drivers.
- Each matrix cell is assigned a score to characterize the relationship between each pressure and threat based on the published scientific literature:
  - 0 indicates no evident link between the pressure and the threat;
  - 1 reflects a direct and well-established link supported by ecological literature or regulatory interpretation;
  - 2 corresponds to an indirect or context-dependent link, where a causal relationship exists but varies by sector, geography, or analytical scale.

For example, satellite accounts related to ammonia ($NH_3$), nitrous oxide ($N_2O$), and land-use expansion are directly linked (score = 1) to IUCN categories 2 (“Agriculture & Aquaculture”) and 9 (“Pollution”).

Given the context-dependent nature of indirect links (score = 2), which makes their quantitative attribution uncertain, we set these entries to zero and restrict the *nSTAR* calculation to direct pressure–threat links (score = 1). Moreover, the matrix does not differentiate the relative contribution of each pressure to a given threat; all linked pressures are assigned equal weight, so it functions solely as a connection matrix. Lastly, some threat categories (e.g., Invasive species, Geological events, Human intrusions) could not be matched to GLORIA satellites, reflecting structural limitations of the database, which captures emissions and land-use pressures but not other biodiversity drivers. Finally, only 104 pressures were kept in the analysis below due to lack of data in the GLORIA database across countries.

### Induced extinction risk by pressures of economic sectors

Using the above described matrix, the method to calculate nSTAR scores presented in this study resembles that of Irwin et al.^16^. From the IUCN Red List database, only those species of class “bird”, “mammal”, and “amphibian” with extinction risk categories “Near Threatened” (NT), “Vulnerable” (VU), “Endangered” (EN), and “Critically Endangered” (CR) are kept. Then a numeric value is assigned to each extinction risk category (NT: $W_{i}=1$, VU: $W_{i}=2$, etc), and a Threat Impact Score (*TS*) to each threat (From the Threats Classification Scheme (Version 3.3) by IUCN: https://www.iucnredlist.org/resources/threat-classification-scheme) for which there is information about its scope and severity as classified in Table 2.

**Table 2:** Summary of IUCN threat impact scores by severity and scope. Each of the 124 classified threats is assigned a specific threat impact score per species depending on the scope (i.e., the proportion of the total population affected) and severity (i.e., the overall declines caused by the threat). For the complete ICUN threat impact score data for all species-threat combination refer to https://www.iucnredlist.org/resources/threat-classification-scheme.

		Severity (Causing or likely to cause)			
		Negligible declines OR No decline	Relatively slow, but significant declines OR fluctuations	Rapid declines (20 - 30% over 10 years or three generations)	Very rapid declines (>30% over 10 years or three generations)
Scope	Affects the minority (< 50%) of the population	0	5	7	24
	Affects the majority (50% - 90%) of the population	0	9	18	52
	Affects the whole (> 90%) population	1	10	24	63

The *nSTAR* indicator is obtained by multiplying the extinction risk category $W_{i}$ of species *i* by the Threat Impact Score $TS_{ij}$ for that species under threat *j*:

1 $$\begin{aligned} nSTAR^{(1)}_{ij} = W_{i} * TS_{ij} \end{aligned}$$

This first matrix ($\mathbf {nSTAR^{(1)}}$) has dimension $4{,}590 \times 124$ (number of species *×* number of threats).

Following Irwin et al^16^., each species–threat pair was allocated to countries based on IUCN data on species’ geographic ranges. We merged the *nSTAR* metric (Eq. 1) with species-range data and assigned a value of zero to threats affecting species not present in a given country. Here the calculation begins to deviate from Irwin et al^16^.. Using the correspondence matrix between IUCN threats and GLORIA environmental pressures, we calculate the *nSTAR* value for each sector–country pair by combining information on the quantity of environmental pressures, the related threats affecting each species, and the intensity and scope of the threat exerted by each sector.

First, to obtain *nSTAR* for each pressure–species pair, we divide the threat–species *nSTAR* by the number of pressures associated with threat *j*. For example, IUCN threat 2.3.3 “Agro industry grazing, ranching or farming” is linked to pressures including $N_{2}O$, $CH_{4}$, $NH_{3}$, and pasture land use. Thus,

2 $$\begin{aligned} \mathbf {nSTAR^{(2)}} = \mathbf {nSTAR^{(1)}} \times \textbf{CM}, \end{aligned}$$

where $\textbf{CM}$ is the conversion matrix (dimension *124 × 104*, threats *×* pressures), with entries $CM_{jk} = 1/N_j$ if pressure *k* is linked to threat *j* and 0 otherwise, $N_j$ being the number of pressures associated with threat *j*, and *×* denotes matrix multiplication. The resulting matrix ($\mathbf {nSTAR^{(2)}}$) has dimension $4{,}590 \times 104$ (species *×* pressures).

We then differentiate between global and local pressures. Global pressures, such as $CO_{2}$ emissions, contribute to extinction risk for species worldwide; local pressures are directly connected to species at their location. We adjust the *nSTAR* metric to account for each species’ habitat surface area:

3 $$\begin{aligned} \mathbf {nSTAR^{(3)}} = \textbf{D}(120*4590) \times \mathbf {nSTAR^{(2)}} \otimes \textbf{PR}, \end{aligned}$$

where $\textbf{D}(120*4590)$ is a matrix composed of 120 diagonal matrices of dimension 4, 590 stacked together, $\textbf{PR}$ is a matrix of dimension $120 * 4{,}590 \times 104$ whose columns equal 1 for global pressures or the species’ surface share within each country for local pressures, and *⊗* denotes element-wise multiplication. The resulting matrix ($\mathbf {nSTAR^{(3)}}$) has dimension $(120 * 4{,}590) \times 104$ (countries *×* species, per pressure).

To attribute an *nSTAR* score to each sector within each country, we multiply the previous result by each sector’s share of pressure within its country. For global pressures, the sector share is computed as a proportion of total global pressure (summing across all sectors in all countries):

4 $$\begin{aligned} \mathbf {nSTAR^{(4)}} = \textbf{D}(120*164*4590) \times \mathbf {nSTAR^{(3)}} \otimes \textbf{PS}, \end{aligned}$$

where $\textbf{D}(120*164*4590)$ is a matrix of 164 diagonal matrices of dimension 4, 590 distributed diagonally 120 times (zeros elsewhere), and $\textbf{PS}$ is a matrix of dimension $(120 * 164 * 4{,}590) \times 104$ whose columns contain country-sector global shares for global pressures or sector shares within each country for local pressures. The resulting matrix ($\mathbf {nSTAR^{(4)}}$) has dimension $(120 * 164 * 4{,}590) \times 104$ (countries *×* sectors *×* species, per pressure).

Finally, scores are aggregated by species to obtain values at the country, sector, and pressure level:

5 $$\begin{aligned} \textbf{S} = \mathbf {nSTAR^{(5)}} = \textbf{I}(120*164 \times 120*164*4590) \times \mathbf {nSTAR^{(4)}}, \end{aligned}$$

where $\textbf{I}(120 \times 164 \times 120 \times 164 \times 4{,}590)$ is a matrix of unit vectors of dimension 4, 590 arranged diagonally. The resulting matrix ($\textbf{S} = \mathbf {nSTAR^{(5)}}$) has dimension $(120 \times 164) \times 104$ (countries *×* sectors, per pressure).

The matrix $\textbf{S}$ contains *nSTAR* scores for each country–sector pair in its rows and each environmental pressure in its columns ($120 \times 164 \times 104$). Scores are also aggregated across pressures to obtain a consolidated vector of total *nSTAR* for computing country net footprints:

6 $$\begin{aligned} \textbf{s} = \textbf{S} \times \textbf{I}, \end{aligned}$$

where $\textbf{I}$ is a unit vector of dimension *104 × 1*. The resulting vector $\textbf{s}$ has dimension (120 * 164) × 1 (countries *×* sectors).

We then define the pressure intensity vector $\textbf{e} = \textbf{s} \oslash \textbf{x}$, where $\textbf{x}$ is the vector of total output per sector in each country.

### Global net footprint

The environmental impact matrix $\textbf{E}$, of dimension (120 * 164) × (120 * 164), records the environmental impact associated with transactions between economic sectors to satisfy final demand. It is computed as:

7 $$\begin{aligned} \textbf{E} = {\hat{\textbf{e}}} \times \textbf{L} \times {\hat{\textbf{f}}} \end{aligned}$$

where $\textbf{L}$ is the Leontief matrix of inter-industry production multipliers of dimensions 19680x19680, and $\textbf{f}$ is the final demand vector of dimensions 19680x1. ${\hat{\textbf{e}}}$ is the pressure intensity diagonal matrix and ${\hat{\textbf{f}}}$ is the world final demand diagonal matrix. Row sums of $\textbf{E}$ equal the total production-based environmental footprint captured by $\textbf{e}$.

The country footprint (*NFP*) of *nSTAR* for each country *c* is computed as $NFP = \text {Production} - \text {Exports} + \text {Imports}$:

8 $$\begin{aligned} \textbf{NFPc} = \textbf{s} \times \mathbf {I_c} - \text {diag}\left( \mathbf {I_c} \times \textbf{E} \times \mathbf {I_{nc}}^{\intercal }\right) + \text {diag}\left( \mathbf {I_{nc}} \times \textbf{E} \times \mathbf {I_c}^{\intercal } \right) \end{aligned}$$

where $\mathbf {I_c}$ is a matrix of dimension $(120 \times 164) \times 164$ with 1 in each row pertaining to the country of the column and 0 otherwise (for the first country, the first column has 1 in rows 1–120 and 0 elsewhere), $\mathbf {I_{nc}}$ is the complementary matrix with 0 in each row pertaining to the country and 1 otherwise, and $\text {diag}(\cdot )$ extracts the diagonal elements of a matrix.

The second term represents exports: pressures occurring domestically but generated for goods and services demanded elsewhere (country sectors $\mathbf {I_{c}}$ satisfying demand from outside the country $\mathbf {I_{nc}}$). The third term represents imports: pressures from non-domestic sectors ($\mathbf {I_{nc}}$) satisfying domestic demand ($\mathbf {I_{c}}$). The resulting vector $\textbf{NFPc}$, of dimension *164 × 1*, contains the net *nSTAR* footprint for each country.

### Sectoral net footprint

The total net footprint (*NFP*) of *nSTAR* for each sector *z* is:

9 $$\begin{aligned} \textbf{NFPs} = \textbf{s} \times \mathbf {I_s} - \text {diag}\left( \mathbf {I_s} \times \textbf{E} \times \mathbf {I_{ns}}^{\intercal }\right) + \text {diag}\left( \mathbf {I_{ns}} \times \textbf{E} \times \mathbf {I_s}^{\intercal } \right) \end{aligned}$$

where $\mathbf {I_s}$ is a matrix of dimension $(120 \times 164) \times 120$ with 1 in each row pertaining to the sector of the column and 0 otherwise (for the first sector, the first column has 1 in rows 1, 165,..., 19561 and 0 elsewhere), and $\mathbf {I_{ns}}$ is the complementary matrix with 0 in each row pertaining to a sector and 1 otherwise.

The second term represents sector exports: pressures occurring within a sector but generated for demand from other sectors (sector $\mathbf {I_{s}}$ satisfying demand from other sectors $\mathbf {I_{ns}}$). The third term represents sector imports: pressures from other sectors ($\mathbf {I_{ns}}$) satisfying demand for the sector’s goods and services ($\mathbf {I_{s}}$).

The resulting vector $\textbf{NFPs}$, of dimension *120 × 1*, contains the net *nSTAR* footprint for each sector.

### Marginal reduction of European extinction risk footprint

Using vector $\textbf{e}$, we calculate the total consumption-based footprint (*FP*) exerted by all countries and sectors:

10 $$\begin{aligned} \textbf{FP} = \textbf{L}^{\intercal } \times \textbf{e} \otimes \textbf{f} \end{aligned}$$

Using Equation 10, we calculate total *nSTAR* footprints and simulate a hypothetical policy targeting a 1% reduction in the combined consumption-based extinction risk footprint of European sectors, by deriving the variation in final demand consistent with this target:

$$\begin{aligned} \Delta \textbf{f} = \Delta \textbf{FP} \oslash (\textbf{L}^{\intercal }\textbf{e}) \end{aligned}$$

The corresponding variation in output is then computed using the classical Leontief equation:

$$\begin{aligned} \Delta \textbf{x} = \textbf{L} \times \Delta \textbf{f} \end{aligned}$$

The 120 GLORIA sectors are then aggregated according to the NACE rev. 2 classification.

### Linking BACH financial data to GLORIA sectoral footprints

To assess the financial implications of the biodiversity-footprint reduction, we linked the sectoral output changes derived from the GLORIA MRIO simulation to financial data from the BACH dataset. The 120 GLORIA sectors were first aggregated to the NACE rev. 2 classification to match the sectoral disaggregation of BACH. For each country–sector pair covered by both datasets, we extracted the baseline interest-payments-to-turnover ratio directly from BACH.

To estimate how this ratio changes following a reduction in sectoral output, we assume that turnover is proportional to output, which is consistent with the fixed-coefficient Leontief framework. Under this assumption, a proportional decline in output translates directly into a proportional decline in turnover. Since nominal interest payments are fixed in the short run, a decline in turnover increases the interest-payments-to-turnover ratio, indicating a higher debt burden relative to revenue. The relative variation in this ratio is:

11 $$\begin{aligned} \frac{r_1 - r_0}{r_0} = \frac{\delta }{1-\delta } \end{aligned}$$

where $r_1 = r_0 / (1-\delta )$ is the new ratio, $r_0$ is the baseline interest-payments-to-turnover ratio from BACH, and $\delta = \Delta x / x$ is the proportional output reduction from the Leontief simulation.

## Supplementary Information

Supplementary Information 1.

Supplementary Information 2.

## Data Availability

Datasets generated in this study and the pressure to threats concordance matrix have been deposited in the following Zenodo repository: https://zenodo.org/records/17908768. GLORIA MRIO is available here. IUCN Biodiversity Threats classification scheme is available here.
