# Supplementary material for: Trade induced extinction risk footprints by European financial institutions
Source: Sci Rep. 2026 Jul 10;16:32131. doi: 10.1038/s41598-026-58778-3 (PMC13661235; doi:10.1038/s41598-026-58778-3)

Supplementary Figures & Tables

Tables

Table S1: All environmental pressures analyzed and their contribution to nSTAR score

**Pressure**	**nSTAR**	**Percentage (%)**
NH3	22412	8.5857
N2O	15541	5.9537
CO2_excl_short_cycle_org_c	15338	5.8759
CO2_org_short_cycle_c	14873	5.6976
CH4	14779	5.6617
PM25_bio	8614	3.2999
Grazed biomass	7778	2.9796
Pastures (Land use)	7778	2.9796
Pastures (PDF)	7778	2.9796
NOx	7655	2.9326
PM25_fossil	7424	2.8439
PM10	7424	2.8439
CO	6252	2.3952
Annual_crops (Land use)	5459	2.0914
Annual_crops (PDF)	5459	2.0914
Permanent_crops (Land use)	5459	2.0914
Permanent_crops (PDF)	5459	2.0914
Fruits	5456	2.0902
Roots and tubers	5456	2.0902
Oil bearing crops	5456	2.0902
Vegetables	5446	2.0861
Straw	5446	2.0861
Rice	5442	2.0847
Sugar crops	5417	2.0753
Tobacco	5361	2.0538
Fibres	5017	1.922
Nuts	4612	1.7668
Wheat	4021	1.5406
Timber (Industrial roundwood)	3756	1.4389
Intensive_forestry (Land use)	3692	1.4142
Intensive_forestry (PDF)	3692	1.4142
Agriculture water stress	2414	0.9248
SO2	2299	0.8806
Extensive_forestry (Land use)	2143	0.821
Extensive_forestry (PDF)	2143	0.821
Captured energy	1939	0.743
Non-agriculture water stress	1064	0.4074
Non-agriculture blue water consumption	1064	0.4074
Structural clays	861	0.3298
Sand gravel and crushed rock for construction	861	0.3298
Gold ores	805	0.3084
Industrial minerals n.e.c	774	0.2965

Continued on next page

**Pressure**	**nSTAR**	**Percentage (%)**
Salt	689	0.2641
Other metal ores	677	0.2593
Specialty clays	624	0.239
Silver ores	582	0.2231
Copper ores	551	0.2111
Iron ores	472	0.1807
Chemical minerals n.e.c.	437	0.1673
Nickel ores	430	0.1649
SF6	417	0.1049
NMVOC	412	0.1578
Ornamental or building stone	396	0.1517
Coal and peat	375	0.1436
Other Bituminous Coal	368	0.1408
Bauxite and other aluminium ores - gross ore	367	0.1407
Industrial sand and gravel	366	0.1403
Zinc ores	356	0.1366
Manganese ores	343	0.1314
Lead ores	317	0.1215
Tin ores	274	0.1051
Titanium ores	251	0.0961
Chromium ores	241	0.0924
Dolomite	238	0.0911
Agriculture blue water consumption	201	0.077
Platinum group metal ores	153	0.0587
Other Sub-Bituminous Coal	153	0.0585
Natural gas	143	0.0548
Crude oil	142	0.0544
Natural gas liquids	135	0.0519
Chalk	112	0.0429
Lignite (brown coal)	109	0.0419
Uranium ores	91	0.0347
Other non-metallic minerals n.e.c.	84	0.0324
Anthracite	71	0.0272
Oil and natural gas	52	0.0198
BC	52	0.0198
OC	52	0.0198
Peat	44	0.0168
Wood fuel and other extraction	34	0.013
Wild fish catch	14	0.0052
All other aquatic animals	14	0.0052
hfc_143a	5	0.0019
hfc_32	5	0.0019
hfc_125	5	0.0019
hfc_134a	5	0.0019
hfc_152a	5	0.0019
hfc_227ea	5	0.0019
hfc_245fa	5	0.0019
hfc_365mfc	5	0.0019
Oil shale and tar sands	5	0.0019
C2F6	5	0.0018
C3F8	5	0.0018
CF4	5	0.0018

Continued on next page

Pressure	nSTAR	Percentage (%)
hfc_23	5	0.0018
hfc_236fa	5	0.0018
C4F10	4	0.0017
Gypsum	1	0.0005
Solid biofuels	1	0.0005
Coking Coal	1	0.0004
C4F8	0	0.0001
hfc_43	0	0.0001
Nuclear	0	0.0001
NF3	0	0.0001

Figures

Figure S1: Country trend of Potentially Diseappeared Fraction (PDF) related to Extensive Agriculture, Intensive Agriculture, Pasture, Extensive Forestry, and Intensive Forestry (2019).

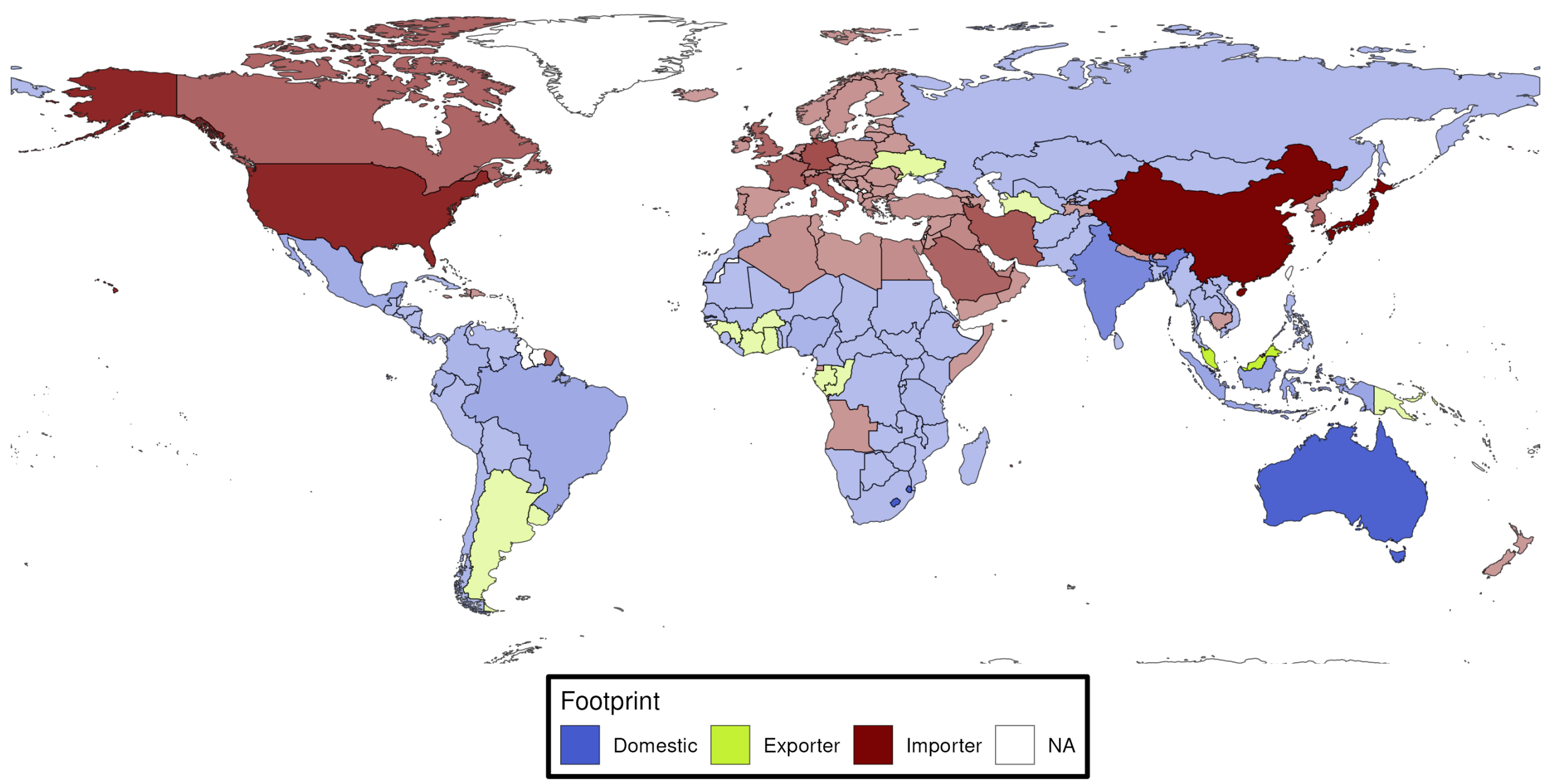

Figure S2: Highest impact economic sectors by country.

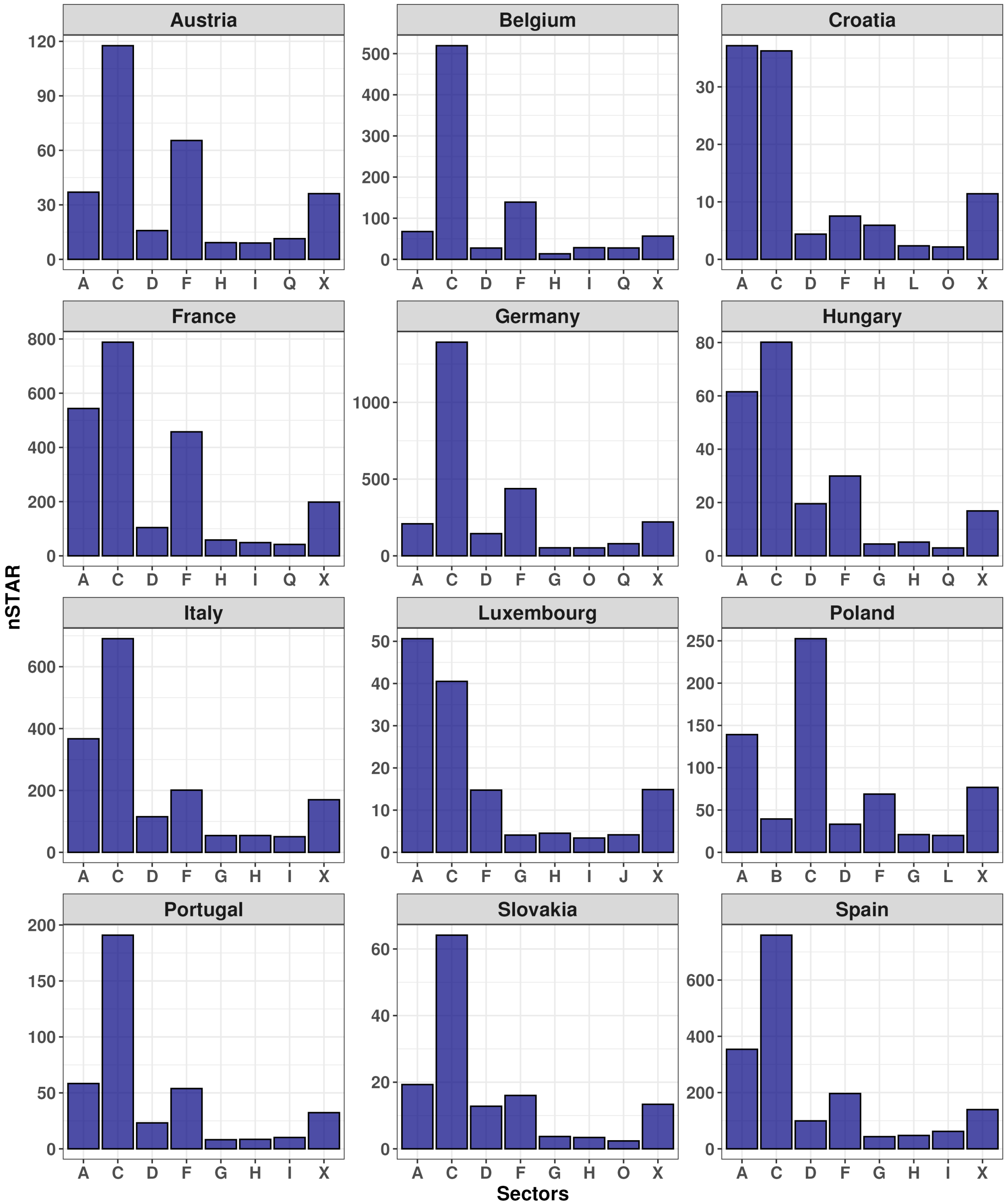

Supplement: Supplementary file 1 [file 41598_2026_58778_MOESM1_ESM.pdf]
