# Supplementary material for: Trade induced extinction risk footprints by European financial institutions
Source: Sci Rep. 2026 Jul 10;16:32131. doi: 10.1038/s41598-026-58778-3 (PMC13661235; doi:10.1038/s41598-026-58778-3)
Supplement: Supplementary file 2 [file 41598_2026_58778_MOESM2_ESM.pdf]

Supplementary material

Correspondence matrix: building literature

We present here the rationale and literature that inform the development of the correspondence matrix between IUCN threats to biodiversity and GLORIA environmental pressures. This was done to enable the integration of extinction risk assessments into environmentally extended input–output (EEIO) models. It supports the application of the STAR (Species Threat Abatement and Restoration) metric by mapping environmental pressure indicators from the GLORIA MRIO database to subcategories from the IUCN Threat Classification Scheme (Version 3.3). The matrix was constructed through a qualitative, expert-based assessment process. Each cell represents one of three correspondence levels: 0 (no plausible causal link), 1 (direct and well-documented link), or 2 (indirect or context-dependent link). The scoring drew on IUCN documentation, relevant ecological literature (e.g. IPBES 2019; Lamb et al. 2021; Mair et al. 2021), and expert judgment. The resulting framework captures plausible associations between sector-level pressures and biodiversity threats.

The matrix is intended to serve as a methodological bridge between environmental accounting data and threat-based biodiversity indicators. It does not quantify impact magnitude but instead provides a structured representation of the presence or absence of potential threat pathways. All IUCN threat subcategories were retained in the structure, including those for which no correspondence was identified with any GLORIA pressure indicator. These appear in the matrix with zero scores throughout.

The extract presented below illustrates a subset of the correspondence matrix, selected to reflect a diversity of link types while remaining concise for reporting purposes. It includes a sample of satellite indicators and threat subcategories. The full matrix comprises 104 GLORIA satellite indicators and 124 IUCN threat subcategories. The complete version of the correspondence matrix is available from the authors upon request.

The justification for the value of each cell in the matrix are the following

- C4F8, C2F6, C3F8, C4F10, C5F12, C6F14, C7F16, CF4, C4F8_DALY, C2F6_DALY, C3F8_DALY, C4F10_DALY, C5F12_DALY, C6F14_DALY, C7F16_DALY, CF4_DALY:
  - Justification: Perfluorocarbons (PFCs) are potent greenhouse gases due to their long lifetimes and strong absorption in the infrared atmospheric window region, resulting in global warming potentials (GWPs) on a 100-yr time horizon of three to four orders of magnitude higher than that of carbon dioxide (CO2). PFCs are considered to have a nearly permanent effect on the Earth's radiative budget, when human time scales are considered. PFCs are included as one of the six classes of greenhouse gases under the Kyoto Protocol to the United Nations Framework Convention on Climate Change (UNFCCC). The most significant emission source of the high molecular weight PFCs are from their use as solvents in electronics and precision cleaning. There are also small niche markets for C4F10 and C6F14 as fire suppressants, The PFCs, which are liquid at room temperature, C5F12- C8F18 are additionally being used in the semiconductor manufacturing industry as heat transfer fluids and in vapor phase reflow soldering. This is why there are direct links to air-borne pollutants, and emissions to the atmosphere. Additionally, there are links to threats of fire suppression for components used for that.
  - Source: IPBES (2019); IUCN (2019); Ivy, Rigby, Baasandorj, Burkholder, and Prinn (2012)
  - Limitation: One of the main sources of emissions is the use of cleaning, solvents in electronics and manufacturing. This is hard to link to IUCN threats as the industry creating the emissions is not clear in the guidelines, we linked the threats due to their pollutant properties.
- hfc_23, hfc_23_DALY:
  - Justification: The gas HFC-23 (trifluoromethane, fluoroform, CHF3) is a powerful greenhouse gas that is formed at the reactor stage of the manufacture of HCFC-22 (chlorodifluoromethane, CHClF2). It is principally used as a refrigerant in several different applications, as a blend component in foam blowing and also as a chemical feedstock for manufacturing synthetic polymers. In

developed countries, the scheduled phase out of HCFC-22 consumption (due to its stratospheric ozone depleting properties) has commenced. This gas was linked to pollution.

- hfc_32, hfc_43, hfc_125, hfc_134a, hfc_143a, hfc_152a, hfc_227ea, hfc_236fa, hfc_245fa, hfc_365mfc, hfc_32_DALY, hfc_43_DALY, hfc_125_DALY, hfc_134a_DALY, hfc_143a_DALY, hfc_152a_DALY, hfc_227ea_DALY, hfc_236fa_DALY, hfc_245fa_DALY, hfc_365mfc_DALY
  - Justification: Halogenated chlorofluorocarbons are used as working fluids in refrigeration. The thermal conductivity of the refrigerants has a very important role in the design of heat exchangers. Research showed that in the UK Refrigerant emissions add 20
  - Source: IPBES (2019); IUCN (2019); Johnson (2011)
- NF3, NF3_DALY
  - Justification: Nitrogen trifluoride (NF3) is greenhouse gas: It is a synthetic chemical produced in industrial quantities. NF3 has a potential greenhouse impact larger than that of the industrialized nations' emissions of PFCs or SF 6, or even that of the world's largest coal-fired power plants. If released, annual production would increase the lower atmospheric abundance by 0.4 ppt. NF3 was previously used in rocket fuel and lasers. Now it is used as plasma etchant and equipment cleaning gas in the semiconductor industry. In this case we did direct links to threats regarding air-borne pollutants and emissions to the atmosphere, but we also included as indirect links industrial development threats from the IUCN because it is used in manufacturing processes and semiconductor.
  - Source: IPBES (2019); IUCN (2019); Prather and Hsu (2008)
- SF6, SF6_DALY
  - Justification: SF6 is one of the most potent greenhouse gases. This gas has high atmospheric stability and ability to trap infrared radiation means it's far more potent at warming the earth's atmosphere than CO2 over longer periods of time. SF6 is used globally in electricity transmission and distribution. Medium- and high-voltage electrical equipment contains SF6 to insulate the live electrical parts and to switch the flow of electrical current on and off. We created direct links to emissions to the atmosphere and air borne pollutants, we included as indirect links threats regarding transportation and transmission if electricity.
  - Source : IPBES (2019); IUCN (2019); Parris (2011)
- CO2_excl_short_cycle_org_c, CO2_org_short_cycle_c, CO2_excl_short_cycle_org_c_DALY, CO2_org_short_cycle_c_DALY:
  - Justification: Compounds resulting from combustion contaminate natural sources by generating emissions into the atmosphere (GHG). The highest emitting sectors include energy, electricity and heat, land-use change and management, road transport, residential buildings, metals, chemicals, enteric fermentation (i.e. livestock rearing), non-residential buildings, oil and gas fugitive emissions, and the waste sector. Contributions of anthropogenic CO2 come from the loss of soil organic matter (SOM) in cultivated soils.
  - Source : IPBES (2019); IUCN (2019); Lamb et al. (2021); Paustian, Six, Elliott, and Hunt (2000); United Nations Environment Programme (2016b)
  - Limitation : The amount of threats and drivers that can generate emissions of CO2 is extensive so priorization of factors was crucial for determining important links
- N2O, N2O_DALY:
  - Justification: Relevant emissions of N2O are directly related to agriculture specifically managing soils and pastures, energy sectors with less influence and chemicals manufacture industries.
  - Source: IPBES (2019); IUCN (2019); Lamb et al. (2021); United Nations Environment Programme (2016b)

- CH4, CH4_DALY:
  - Justification: Emissions from agriculture are mainly comprised of CH4 emissions from enteric fermentation (e.g. cattle and sheep digestion), rice cultivation, manure management, biomass burning and fertiliser application.
  - Source: IPBES (2019); IUCN (2019); Lamb et al. (2021); United Nations Environment Programme (2016b)
- BC, OC, BC_DALY, OC_DALY:
  - Justification: Black Carbon and Primary Organic Carbon (OC) emissions come from fossil fuels, biofuels, open biomass burning, and burning of urban waste. Previous inventories of black and organic carbon have assigned emission factors on the basis of fuel type and economic sector alone. We created direct links to the oil industry, air borne pollutants and emissions to the atmosphere
  - Source: Bond et al. (2004); IPBES (2019); IUCN (2019)
- CO, CO_DALY:
  - Justification: The sources of CO are cars, trucks or machinery that burn fossil fuels. A variety of items household areas such as unvented kerosene and gas space heaters, leaking chimneys and furnaces, as well as gas stoves.
  - Source: IPBES (2019); IUCN (2019); United Nations Environment Programme (2016b); US Environmental Protection Agency (2022)
- NH3, NH3_DALY:
  - Justification: Emissions of ammonia considered include the : (1) farmland ecosystem (soil and nitrogen-fixing plant emission, fertilizer application and crop residue compost), (2) livestock wastes (free-range, intensive and grazing rearing systems), (3) biomass burning (forest and grass fires, crop residue burning and fuel wood combustion), (4) excrement of rural populations; 5) chemical industry (ammonia synthesis and nitrogen fertilizer production), (6) waste disposal (wastewater and solid waste treatment), and (7) traffic sources. Even if they specify a combustion process, the degradation and oxidation of ammonia by these activities reach the air and contributes to emissions to the atmosphere.
  - Source : Clarisse, Clerbaux, Dentener, Hurtmans, and Coheur (2009); Huang et al. (2012); IPBES (2019); IUCN (2019); Lesmana et al. (2019); United Nations Environment Programme (2016b)
  - Limitations: understanding other levels of pollution; most research is linked to livestock and agriculture
- NMVOC, NMVOC_DALY:
  - Justification: NMVOC are non-methane volatile organic compounds, their emission is linked to burning fuel, combustion from logging and woodfire for energy production, it is used for manufacturing and in several industrial procedures. This is why is linked to industrial area development, logging and harvesting wood for combustion and energy sector related threats.
  - Source: IPBES (2019); IUCN (2019); Sun et al. (2018); United Nations Environment Programme (2016b)
- NOx, NOx_DALY:
  - Justification: Emissions of nitrogen oxides (NOx) are associated with roads transport, energy production, commercial and household activities.
  - Source: IPBES (2019); IUCN (2019); United Nations Environment Programme (2016a, 2016b) EEA, 2014
  - Limitations: Level of impact of each industry that produces Nox

- PM25_bio, PM25_bio_DALY:
  - Justification: PM2.5_bio refers to particles that are produced from biological sources, such as dust and pollen, as well as emissions from cooking and burning of biomass, including wood, crop residues, and dung. These particles are also sometimes referred to as organic carbon or biogenic PM2.5. Even if they come from organic sources, particulate matter from this size is usually linked to increased pollution specially from burning biomass. Take into accounts challenges distinguishing PM from organic sources or non-organic sources, in any case this particulate matter contributes to air borne pollution especially from burning biomass
  - Source : Brook et al. (2013); Di et al. (2017); IPBES (2019); IUCN (2019); Lelieveld and Evans (2015); Menon et al. (2012); United Nations Environment Programme (2016b); United States Environmental Protection Agency (2016)
- PM25_fossil, PM25_fossil_DALY:
  - Justification: Emissions of particles into the atmosphere (PM2.5 - PM10) are related to oil producing in least developing countries, it is linked to emissions due to residential energy use and transportation, such as heating and cooking, vehicles and traffic.
  - Source : IPBES (2019); IUCN (2019); Munir (2017); United Nations Environment Programme (2016b)
- PM10, PM10_DALY:
  - Justification: Emissions of particles into the atmosphere (PM2.5 - PM10) are related to oil producing in least developing countries, it is linked to emissions due to residential energy use and transportation, such as heating and cooking, vehicles and traffic.
  - Source : IPBES (2019); IUCN (2019); Munir (2017); United Nations Environment Programme (2016b)
- SO2, SO2_DALY:
  - Justification: Emissions of SO2 from the combustion and oxidation of fuels and other materials are due to industry and shipping. It also is one of the major contributors to acid rain.
  - Source : IPBES (2019); IUCN (2019); Klimont, Smith, and Cofala (2013); United Nations Environment Programme (2016b)
  - Limitations: It can be linked to shipping but is hard to link the levels of transportation with the threats proposed by the IUCN
- Rice, Wheat:
  - Justification: Land used for agriculture is connected to threats involving agricultural expansion. Habitat loss and degradation are the most direct threats to biodiversity (terrestrial mammals and birds) and about the 80% of threatened terrestrial bird and mammal species are imperilled by agriculturally driven habitat loss, other important drivers include at the same time fragmentation of natural habitats, this is why there is an indirect link.
  - Source : Tilman, Balzer, Hill, and Befort (2011)
- Cereals n.e.c., Other crops n.e.c, Spice - beverage - pharmaceutical crops:
  - Justification: Land used for agriculture is connected to threats involving agricultural expansion. Habitat loss and degradation are the most direct threats to biodiversity (terrestrial mammals and birds) and about the 80% of threatened terrestrial bird and mammal species are imperilled by agriculturally driven habitat loss, other important drivers include at the same time fragmentation of natural habitats, this is why there is an indirect link. Primary crops and agriculture in general have proven to be one of the major threats to biodiversity and conservation priority areas, due to the expansion of land use for cultivation. Additionally, nutrient pollution have long damaged

freshwater biodiversity and are increasing affecting marine habitats, with over 500 ‘dead zones’ of oxygen-depleted water around the world. Similar impacts are linked to other pesticides, fungicides and herbicides, often acting in combination.

  - Source : Dudley and Alexander (2017); IPBES (2019); IUCN (2019); Tilman et al. (2011); Veach, Moilanen, and Di Minin (2017)
  - Justification: Rice, Wheat + Tobacco and others

- Tobacco, Roots and tubers, Sugar crops, Pulses, Nuts, Oil bearing crops, Vegetables, Fruits, Fibres, Straw, Other crop residues (sugar and fodder beet leaves etc), Fodder crops (including biomass harvest from grassland), Grazed biomass:
  - Justification: Primary crops and agriculture in general have proven to be one of the major threats to biodiversity and conservation priority areas, due to the expansion of land use for cultivation. Additionally, nutrient pollution have long damaged freshwater biodiversity and are increasing affecting marine habitats, with over 500 ‘dead zones’ of oxygen-depleted water around the world. Similar impacts are linked to other pesticides, fungicides and herbicides, often acting in combination.
  - Source : Dudley and Alexander (2017); IPBES (2019); IUCN (2019); Veach et al. (2017)

- Timber (Industrial roundwood), Wood fuel and other extraction:
  - Justification: Extraction of wood, logging and harvesting roundwood in forest lands or other areas are linked to the threats relating to logging and rates of extraction of living materials. For emissions generated in this case there are links from these stressors to the contamination it creates, such as emissions to the atmosphere and air borne pollution due to the release of gases in burning. Recent studies in wood extraction, transformation and transportation have showed that on-road transportation of wood fuel is one of the major components influencing GHG emissions (specially CO2), without taking into account the regular emissions for energy consumption.
  - Source : IPBES (2019); IUCN (2019)Pierobon et al, 2015

- Wild fish catch, All other aquatic animals, Aquatic plants:
  - Justification: Annual global fish catches have reached 90 million metric tons per year; it has been observed that fisheries are overfished and the trophic level of fish caught worldwide has declined substantially. Additionally, aquaculture has been cited to increase the seafood supply however it has not shown to be sufficient for the global demand, so aquaculture is not substituting wild fisheries and markets have heavy dependence on wild fish inputs. In this case the links we made where direct for threats regarding extraction and harvesting of marine resources and indirect links were made to aquaculture due to the increase of fish farming.
  - Source : Goldburg and Naylor (2005); IUCN (2019)

- Iron ores, Silver ores, Bauxite and other aluminium ores - gross ore, Gold ores, Chromium ores, Copper ores, Manganese ores, Other metal ores, Nickel ores, Lead ores, Platinum group metal ores, Tin ores, Titanium ores, Uranium ores, Zinc ores, Ornamental or building stone, Chalk, Dolomite, Limestone, Natural gas, Natural gas liquids, Oil shale and tar sands:
  - Justification: The ore industry, and quarrying activities are linked to threats regarding extraction of ores, quarrying, mining and land degradation but the extraction of these materials is also linked to different types of pollution. In this case it is included run-off and seepage from mining, meaning all type of water-borne pollutants that can potentially contaminate bodies of water. Furthermore, the dominant emissions generated from material handling, windbox exhaust, discharge end, and cold screen are particulate emissions mainly releasing iron oxides, sulphur oxides, calcium oxides, hydrocarbons, carbonaceous compounds, and chlorides. Emission of particulate matter, carbon monoxide, sulphur dioxide, organic compounds and other pollutants originate from several operations in the coking plants. Emissions from the blast furnace are generated from the top, in the

casting stages, by drilling and plugging the taphole. In this particular case, the iron and steel industry causes significant effects on environmental media: air -emissions of SO2, NO x, CO, H2S, PAH, lead, Ni, As, Cd, Cr, Cu, Zn, Se, Hg, PM, etc.; water - process water with organic matter, oil, metals, suspended solids, benzene, phenol, acids, sulphides, sulphates, ammonia, cyanides, thiocyanates, thiosulfates, fluorides (scrubber effluent); soil - slag, sludge, sulphur compounds, heavy metals, oil and grease residues, salts.

  - Source : Gavel et al. (2021); IPBES (2019); IUCN (2019); United Nations Environment Programme (2016b); Zhu, Tian, and Hao (2020)
  - Limitations: Each ore industry can create specific impacts regarding its extraction, agglomeration, transformation and even transportation. In this case the links created show a global approach due to the tendency of these activities to contaminate however for understanding quantitatively the links more research must be done.

- Fertilizer minerals n.e.c.:
  - Justification: Fertilizers are known to be heavy pollutants, especially N-based enriched compounds due to its tendency to provide nitrogen into the soil, and increase eutrophication.
  - Source : IPBES (2019); IUCN (2019); Koolen and Rothenberg (2019)
  - Limitations: N.E.C (Not Elsewhere Classified) are difficult to link because there is no enough information about their origin and they are highly dependent to the region/place so there is no good resolution for understanding links with biodiversity threats
- Chemical minerals n.e.c.:
  - Justification: In this case chemicals build up from minerals are linked to mineral extraction but also to different kind of pollution.
  - Source : IPBES (2019); IUCN (2019)
  - Limitations: N.E.C (Not Elsewhere Classified) are difficult to link because there is no enough information about their origin and they are highly dependent to the region/place so there is no good resolution for understanding links with biodiversity threats
- Industrial minerals n.e.c:
  - Justification: In this case, industrial minerals are linked to the mining sector but it has direct links with industrial effluents, run-off, water-borne and air-borne pollution.
  - Source : IPBES (2019); IUCN (2019)
  - Limitations: N.E.C (Not Elsewhere Classified) are difficult to link because there is no enough information about their origin and they are highly dependent to the region/place so there is no good resolution for understanding links with biodiversity threats
  - Justification: Salt exploitation is linked to extraction of minerals, mining, and land degradation due to its exploitation. It is linked indirectly to emissions of gases to the atmosphere due to land degradation and drillings.
  - Source : IPBES (2019); IUCN (2019)
- Gypsum, Structural clays, Specialty clays, Industrial sand and gravel, Sand gravel and crushed rock for construction, Other non-metallic minerals n.e.c.:
  - Justification: Building industries are responsible of gypsum, clay and lime exploitation so they are linked to threats of mining activities. Literature has shown that exploitation of these minerals, and activities done by the building industries, are responsible for carbon emissions to the atmosphere. So, there are direct links to air borne pollution and emissions of GHG into the atmosphere.
  - Source : Fořt and Černý (2018); IPBES (2019); IUCN (2019)
- Lignite (brown coal), Other Sub-Bituminous Coal:

- Justification: Industry of coal is linked to quarrying and it has shown multiple environmental problems (i.e., water, soil, and air pollution). In the coal industry air-staged combustion influences considerably NOx emissions from the combustion of anthracite coal.
- Source : Chen et al. (2014); Fan, Lin, Li, Kuang, and Zhang (2009); IPBES (2019); IUCN (2019)

• Anthracite

- Justification : Ref Line 91 + The extraction of raw materials, consisting in biomass, fossil fuels, industrial minerals, ores, etc is linked to the specific threat, mining, oil drilling, fishing, etc.. Therefore, these satellites are directly linked to the extraction of nature inputs from the environment.

• Coking Coal, Other Bituminous Coal:

- Justification: Industry of coal is linked to quarrying and it has shown multiple environmental problems (i.e., water, soil, and air pollution). In the coal industry air-staged combustion influences considerably NOx emissions from the combustion of anthracite coal. + The extraction of raw materials, consisting in biomass, fossil fuels, industrial minerals, ores, etc is linked to the specific threat, mining, oil drilling, fishing, etc. Therefore, these satellites are directly linked to the extraction of nature inputs from the environment.
- Source : Chen et al. (2014); Fan et al. (2009); IPBES (2019); IUCN (2019)

• Peat:

- Justification: Peatlands are extremely important ecosystems being extracted, they are net sinks that store carbon and their constant extraction increases CO2 emissions, they are non-renewable sources of energy and are currently leading to climate change.
- Source : IPBES (2019); IUCN (2019); Worrall et al. (2010)

• Crude oil:

- Justification: Production of Petroleum comes from oil drilling extraction; In general petroleum industry accounts for multiple types of pollution. These industries release pollutants such as Volatile Organic Compounds, greenhouse gases and particulate matter, from various parts of their operations. Soil pollution is extremely important too, coming from the extraction of the oil or from oil spills or leakage in pipelines. Research has even showed how noise and light pollution currently affects biodiversity in exploration sites, current threats and drivers include habitat loss and fragmentation due to fossil fuels industries.
- Source : Harfoot et al. (2018); IPBES (2019); IUCN (2019); Jones, Pejchar, and Kiesecker (2015); Ragothaman and Anderson (2017); United Nations Environment Programme (2016b) Ogri, 2001

• Annual_crops, Permanent_crops:

- Justification from Rice and Toboacco, + Crops and agriculture in general have proven to be one of the major threats to biodiversity and conservation priority areas, due to the expansion of land use for cultivation. Additionally, nutrient pollution has long damaged freshwater biodiversity and are increasing affecting marine habitats, with over 500 'dead zones' of oxygen-depleted water around the world. Similar impacts are linked to other pesticides, fungicides and herbicides, often acting in combination.

• Female, Male, Employment_skill_high, Employment_skill_middle, Employment_skill_low:

- Justification: Employment accounts for satellites comprise employment, gender, skills, and other type of indicators that are hardly linked to a threat. They can be associated to the impacts of jobs (ex: Work and other human activities from IUCN, Emissions of CO2 to the atmosphere from IPBES) but it will not discriminate among indicators of satellites.
- Source : IPBES (2019); IUCN (2019); United Nations Environment Programme (2016b)

- Annual_crops, Permanent_crops, Annual_crops, Permanent_crops:
  - Ref. Line 74 / Ref. Line 79 /
  - Justification: Crops and agriculture in general have proven to be one of the major threats to biodiversity and conservation priority areas, due to the expansion of land use for cultivation. Additionally, nutrient pollution has long damaged freshwater biodiversity and are increasing affecting marine habitats, with over 500 'dead zones' of oxygen-depleted water around the world. Similar impacts are linked to other pesticides, fungicides and herbicides, often acting in combination.
  - Source: Dudley and Alexander (2017); IPBES (2019); IUCN (2019); Veach et al. (2017)
- Intensive_forestry, Extensive_forestry, Intensive_forestry, Extensive_forestry:
  - Justification: Extraction of wood, logging and harvesting roundwood in forest lands or other areas are linked to the threats relating to logging and rates of extraction of living materials. For forestry land change threats are related to logging practices and their impact to biodiversity. Both type of forestry systems release CO2 to the atmosphere, but worst outcomes regarding emissions and land degradation. Here we take into account intensive forestry as logging and wood plantations, localised extraction and extensive as the extraction and logging of wood from natural places or mosaic patches hosting natural forest and planted trees. This changes the IUCN threat linked but not the type of Driver of change from the IPBES
  - Source : Griscom, Goodman, Burivalova, and Putz (2018); IPBES (2019); IUCN (2019)
- Urban:
  - Justification: Urban development is linked to residential and commercial development as it describes the expansion of land use for this type of economic sector
- Coal and peat, Oil and natural gas, Nuclear, Solid biofuels, Captured energy, Heat:
  - This section corresponds to energy accounts, so it includes directly the stockage, transport and use of energy (emissions included)
  - Source : IPBES (2019); IUCN (2019); United Nations Environment Programme (2016b)
- Pastures:
  - Justification: Pastures are directly linked to livestock and farming threats, additionally emissions of ammonia considered threats to biodiversity loss include the : (1) farmland ecosystem (soil and nitrogen-fixing plant emission, fertilizer application and crop residue compost), (2) livestock wastes (free-range, intensive and grazing rearing systems)
  - Source : Clarisse et al. (2009); Huang et al. (2012); IPBES (2019); IUCN (2019); Lesmana et al. (2019); United Nations Environment Programme (2016b)
- Agriculture water stress, Agriculture blue water consumption, Non-agriculture water stress, Non-agriculture blue water consumption:
  - Justification: The impacts on the hydrological system as a whole includes physical impacts such as drainage and physical modification of river channels and modification of the catchment as well as nutrient, particulate and biocide pollution. In this case, water stress influences the direct links to threats. Definition: Agricultural water stress occurs when the demand for water in the agricultural sector exceeds the available supply. This type of water stress is particularly relevant in regions where agriculture is a dominant economic activity and where water resources are limited. Agricultural water stress can lead to reduced crop yields, increased use of groundwater resources, and competition for water among different sectors, such as municipal and industrial users.
  - Note: Non-agricultural water stress, occurs when the demand for water in non-agricultural sectors, such as municipal, industrial, and domestic users, exceeds the available supply. This type of water stress is more common in urban areas where there is high population density, rapid urbanization, and water demand from various sectors. Non-agricultural water stress can lead to water shortages, increased water prices, and conflicts over water resources among different users.

- Limitations: Depending on the type of activity that is disturbing the hydrological regime it can be linked to abstraction of surface water for commercial, housing or industrial purposes. Direct links were established for all usage except agriculture.
- Source : IPBES (2019); IUCN (2019); Moss (2008)

References

Bond, T., Streets, D., Yarber, K., Nelson, S., Woo, J., & Klimont, Z. (2004). A technology-based global inventory of black and organic carbon emissions from combustion. *Journal of Geophysical Research: Atmospheres*, *109*(D14).

Brook, J., Setton, E., Seed, E., Shooshtari, M., Doiron, D., & Martin, R. (2013). Did the vancouver 2010 winter olympics lead to population health benefits? impacts of a mass participation event. *Canadian Journal of Public Health*, *104*(2), e98–e103. doi: 10.17269/cjph.104.3668

Chen, J., Liu, G., Kang, Y., Wu, B., Sun, R., Zhou, C., & Wu, D. (2014). Coal utilization in china: environmental impacts and human health. *Environmental Geochemistry and Health*, *36*(4), 735–753.

Clarisse, L., Clerbaux, C., Dentener, F., Hurtmans, D., & Coheur, P. (2009). Global ammonia distribution derived from infrared satellite observations. *Nature Geoscience*, *2*(7), 479–483.

Di, Q., Dai, L., Wang, Y., Zanobetti, A., Choirat, C., Schwartz, J., & Dominici, F. (2017). Association of short-term exposure to air pollution with mortality in older adults. *JAMA*, *318*(24), 2446–2456. doi: 10.1001/jama.2017.17923

Dudley, N., & Alexander, S. (2017). Agriculture and biodiversity: a review. *Biodiversity*, 1–5. doi: 10.1080/14888386.2017.1351892

Fan, W., Lin, Z., Li, Y., Kuang, J., & Zhang, M. (2009). Effect of air-staging on anthracite combustion and nox formation. *Energy & Fuels*, *23*(1), 111–120.

Fořt, J., & Černý, R. (2018). Carbon footprint analysis of calcined gypsum production in the czech republic. *Journal of Cleaner Production*, *177*, 795–802.

Gavel, D., Adema, A., van der Stel, J., Peeters, T., Sietsma, J., Boom, R., & Yang, Y. (2021). A comparative study of pellets, sinter and mixed ferrous burden behaviour under simulated blast furnace conditions. *Ironmaking & Steelmaking*, *48*(4), 359–369.

Goldburg, R., & Naylor, R. (2005). Future seascapes, fishing, and fish farming. *Frontiers in Ecology and the Environment*, *3*(1), 21–28.

Griscom, B., Goodman, R., Burivalova, Z., & Putz, F. (2018). Carbon and biodiversity impacts of intensive versus extensive tropical forestry. *Conservation Letters*, *11*(1), e12362.

Harfoot, M., Tittensor, D., Knight, S., Arnell, A., Blyth, S., Brooks, S., & Burgess, N. (2018). Present and future biodiversity risks from fossil fuel exploitation. *Conservation Letters*, *11*(4), e12448.

Huang, X., Song, Y., Li, M., Li, J., Huo, Q., Cai, X., & Zhang, H. (2012). A high-resolution ammonia emission inventory in china. *Global Biogeochemical Cycles*, *26*(1).

IPBES. (2019). *Global assessment report on biodiversity and ecosystem services of the intergovernmental science-policy platform on biodiversity and ecosystem services.* Bonn, Germany: IPBES secretariat. Retrieved from `https://doi.org/10.5281/zenodo.3831673` doi: 10.5281/zenodo.3831673

IUCN. (2019). *The iucn red list of threatened species.*

Ivy, D., Rigby, M., Baasandorj, M., Burkholder, J., & Prinn, R. (2012). Global emission estimates and radiative impact of c4f10, c5f12, c6f14, c7f16 and c8f18. *Atmospheric Chemistry and Physics*, *12*(16), 7635–7645.

Johnson, E. (2011). Air-source heat pump carbon footprints: Hfc impacts and comparison to other heat sources. *Energy Policy*, *39*(3), 1369–1381.

Jones, N., Pejchar, L., & Kiesecker, J. (2015). The energy footprint: how oil, natural gas, and wind energy affect land for biodiversity and the flow of ecosystem services. *BioScience*, *65*(3), 290–301.

Klimont, Z., Smith, S., & Cofala, J. (2013). The last decade of global anthropogenic sulfur dioxide: 2000–2011 emissions. *Environmental Research Letters*, *8*(1), 014003. doi: 10.1088/1748-9326/8/1/014003

Koolen, C., & Rothenberg, G. (2019). Air pollution in europe. *ChemSusChem*, *12*(1), 164–172.

Lamb, W. F., Wiedmann, T., Pongratz, J., Andrew, R., Crippa, M., Olivier, J. G., ... House, J. (2021). A review of trends and drivers of greenhouse gas emissions by sector from 1990 to 2018. *Environmental*

research letters, *16*(7), 073005.

Lelieveld, J., & Evans, J. S. (2015). The contribution of outdoor air pollution sources to premature mortality on a global scale. *Nature*, *525*(7569), 367–371.

Lesmana, H., Zhang, Z., Li, X., Zhu, M., Xu, W., & Zhang, D. (2019). Nh3 as a transport fuel in internal combustion engines: a technical review. *Journal of Energy Resources Technology*, *141*(7).

Menon, S., Unger, N., Koch, D., Francis, J., Garrett, T., & Sednev, I. (2012). Aerosol climate impact due to black carbon in snow. *Science*, *337*(6094), 834–837. doi: 10.1126/science.1223447

Moss, B. (2008). Water pollution by agriculture. *Philosophical Transactions of the Royal Society B: Biological Sciences*, *363*(1491), 659–666.

Munir, S. (2017). Analysing temporal trends in the ratios of pm2.5/pm10 in the uk. *Aerosol and Air Quality Research*, *17*(1), 34–48.

Parris, K. (2011). Impact of agriculture on water pollution in oecd countries: recent trends and future prospects. *International Journal of Water Resources Development*, *27*(1), 33–52.

Paustian, K., Six, J., Elliott, E., & Hunt, H. (2000). Management options for reducing co2 emissions from agricultural soils. *Biogeochemistry*, *48*, 147–163.

Prather, M. J., & Hsu, J. (2008). Nf3, the greenhouse gas missing from kyoto. *Geophysical Research Letters*, *35*(12).

Ragothaman, A., & Anderson, W. (2017). Air quality impacts of petroleum refining and petrochemical industries. *Environments*, *4*(3), 66.

Sun, W., Shao, M., Granier, C., Liu, Y., Ye, C., & Zheng, J. (2018). Long-term trends of anthropogenic so2, nox, co, and nmvocs emissions in china. *Earth's Future*, *6*(8), 1112–1133.

Tilman, D., Balzer, C., Hill, J., & Befort, B. L. (2011). Global food demand and the sustainable intensification of agriculture. *Proceedings of the National Academy of Sciences*, *108*(50), 20260–20264.

United Nations Environment Programme. (2016a). *Geo-6 regional assessment for africa.* (Retrieved from `https://www.unep.org/resources/assessment/geo-6-regional-assessment-africa`)

United Nations Environment Programme. (2016b). *Geo-6 regional assessment for north america.* (Retrieved from `https://wedocs.unep.org/handle/20.500.11822/7611`)

United States Environmental Protection Agency. (2016). *Particulate matter (pm) basics.* (Retrieved from `https://www.epa.gov/pm-pollution/particulate-matter-pm-basics`)

US Environmental Protection Agency. (2022). *Basic information about carbon monoxide (co) outdoor air pollution.* (Available via `https://www.epa.gov/co-pollution/basic-information-about-carbon-monoxide-co-outdoor-air-pollution`)

Veach, V., Moilanen, A., & Di Minin, E. (2017). Threats from urban expansion, agricultural transformation and forest loss on global conservation priority areas. *PLoS One*, *12*(11), e0188397.

Worrall, F., Chapman, P., Holden, J., Evans, C., Artz, R., Smith, P., & Grayson, R. (2010). *Peatlands and climate change. scientific review* (Tech. Rep.). David Attenborough Building Iucn, Pembroke St., Cambridge: IUCN UK.

Zhu, C., Tian, H., & Hao, J. (2020). Global anthropogenic atmospheric emission inventory of twelve typical hazardous trace elements, 1995–2012. *Atmospheric Environment*, *220*, 117061.
